# Identification of the potential association between SARS-CoV-2 infection and acute kidney injury based on the shared gene signatures and regulatory network

**DOI:** 10.1186/s12879-023-08638-6

**Published:** 2023-10-03

**Authors:** Xue Zhou, Ning Wang, Wenjing Liu, Ruixue Chen, Guoyue Yang, Hongzhi Yu

**Affiliations:** 1 Department of Nephrology, Haihe Hospital, Tianjin University, 890 Jingu Road, Jinnan District, Tianjin, 300350 China; 2 Department of Nephrology, Tianjin Haihe Hospital, Tianjin, 300350 China; 3 Haihe Clinical School, Tianjin Medical University, Tianjin, 300350 China; 4 Tianjin Institute of Respiratory Diseases, Tianjin, 300350 China; 5 The Third Central Hospital of Tianjin, 83 Jintang Road, Hedong District, Tianjin, 300170 China; 6 Tianjin Haihe Hospital, Tianjin, 300350 China; 7 Department of Respiratory Medicine, Tianjin Haihe Hospital, 890 Jingu Road, Jinnan District, Tianjin, 300350 China

**Keywords:** COVID-19, Acute kidney injury, Differentially expressed genes, Biomarker, Pathogenesis

## Abstract

**Background:**

The severe acute respiratory syndrome coronavirus 2 (SARS-CoV-2) infection is identified as the cause of coronavirus disease 2019 (COVID-19) pandemic. Acute kidney injury (AKI), one of serious complications of COVID-19 infection, is the leading contributor to renal failure, associating with high mortality of the patients. This study aimed to identify the shared gene signatures and construct the gene regulatory network between COVID-19 and AKI, contributing to exploring the potential pathogenesis.

**Methods:**

Utilizing the machine learning approach, the candidate gene signatures were derived from the common differentially expressed genes (DEGs) obtained from COVID-19 and AKI. Subsequently, receiver operating characteristic (ROC), consensus clustering and functional enrichment analyses were performed. Finally, protein-protein interaction (PPI) network, transcription factor (TF)-gene interaction, gene-miRNA interaction, and TF-miRNA coregulatory network were systematically undertaken.

**Results:**

We successfully identified the shared 6 candidate gene signatures (RRM2, EGF, TMEM252, RARRES1, COL6A3, CUBN) between COVID-19 and AKI. ROC analysis showed that the model constructed by 6 gene signatures had a high predictive efficacy in COVID-19 (AUC = 0.965) and AKI (AUC = 0.962) cohorts, which had the potential to be the shared diagnostic biomarkers for COVID-19 and AKI. Additionally, the comprehensive gene regulatory networks, including PPI, TF-gene interaction, gene-miRNA interaction, and TF-miRNA coregulatory networks were displayed utilizing NetworkAnalyst platform.

**Conclusions:**

This study successfully identified the shared gene signatures and constructed the comprehensive gene regulatory network between COVID-19 and AKI, which contributed to predicting patients’ prognosis and providing new ideas for developing therapeutic targets for COVID-19 and AKI.

**Supplementary Information:**

The online version contains supplementary material available at 10.1186/s12879-023-08638-6.

## Introduction

Currently, the severe acute respiratory syndrome coronavirus 2 (SARS-CoV-2) is identified as the cause of the massive outbreak of coronavirus disease 2019 (COVID-19), which is rapidly evolving and expanding worldwide leading to the infection of many patients present with severe symptoms [1]. Since the World Health Organization (WHO) proclaimed that COVID-19 entered a period of global epidemic on March 11, 2020, which has posed significant challenges to society and healthcare systems [2]. According to the report from WHO, the number of the patients already infected COVID-19 exceeds 600 million and the fatal cases reaches 6.5 million as of 1 October 2022. Although the infection of SARS-CoV-2 virus mainly causes an acute respiratory symptom, growing evidence substantiates that many other organs apart from the lung have been affected by different degrees of virus injury [3]. Significant efforts have been devoted to explore the pathogenesis of COVID-19, of which viral destruction, inflammatory storm, and the activation of coagulation and complement systems may be regarded as the critical factors in disease development and progression [4].

Acute kidney injury (AKI), the prevalent clinical pathological syndrome, is characterized by high morbidity and mortality [5–7]. AKI occurs frequently in patients with critically ill conditions with renal replacement therapy (RRT), leading to poor prognosis [8]. Among the COVID-19 patients, renal impairment is frequent, of which more than 40% cases present with abnormal proteinuria [9]. Moreover, AKI commonly occurs in severe cases with COVID-19, which is recognized as a marker predicting the severity of current disease associated with unfavorable outcomes [10]. Studies have found a high incidence of AKI in patients with COVID-19. According to the study from 13 hospitals in New York, AKI occurred in 1993 (36.6%) of 5449 COVID-19 patients [11]. Currently, no effective therapeutic intervention is available for COVID-19-related AKI. Moreover, AKI occurrence is significantly correlated with the odds of deaths in hospitalized patients with COVID-19 [12–14]. Therefore, it is urgent to early recognize patients with AKI risk, and to guide preventive and therapeutic strategies for avoiding and arresting the occurrence and progression of AKI in patients with COVID-19.

In this viewpoint, we concentrated on investigating the shared key gene signatures between COVID-19 and AKI, and understanding the potential pathogenesis. Gene Expression Omnibus (GEO) is an international public repository of microarray chips, second-generation sequencing, and other forms of high-throughput genomic data uploaded by researchers worldwide. The dataset can be obtained from GEO database [15]. The COVID-19 dataset GSE157103 and AKI dataset GSE30718 were utilized to identify COVID-19-DEGs and AKI-DEGs, respectively. By taking the intersection of COVID-19-DEGs and AKI-DEGs, 14 shared DEGs were successfully determined between COVID-19 and AKI. Furthermore, we ultimately screened out 6 key gene signatures from the 14 shared DEGs for the model construction via the least absolute shrinkage and selection operator (LASSO) method. Then, receiver operating characteristic (ROC) analysis [16] was utilized to assess the predictive efficiency in COVID-19 and AKI cohorts, respectively.

The concept of precision medicine promotes the subgroups analysis in a single study, with different subgroups having different pathogenic mechanisms and clinical prognostic characteristics. Similar subgroup analyses had performed in the previous studies [17]. We investigated the COVID-19 subgroups divided by these key gene signatures and clarified the individual difference in COVID-19 patients. Finally, we conducted the comprehensive gene regulatory networks analyses, such as PPI, transcription factor (TF)-gene interaction, gene-miRNA interaction, and TF-miRNA coregulatory networks utilizing NetworkAnalyst platform for systematically investigating the potential gene regulatory mechanisms. This study identifies the shared gene signatures and explored the gene regulatory networks between COVID-19 and AKI, which may contribute to better predicting the risk of AKI and developing a series of strategies for clinical management in the patients with COVID-19 and AKI.

## Materials and methods

### Data retrieval

To explore the common genetic interrelations between COVID-19 and AKI, two datasets GSE157103 and GSE30718 were retrieved from GEO database (http://www.ncbi.nlm.nih.gov/geo/). The processing platform for GSE157103 was GPL24676 (Illumina NovaSeq 6000), which provided 100 COVID-19 samples and 26 control samples. The processing platform for GSE30718 was GPL570 (Affymetrix Human Genome U133 Plus 2.0 Array), which provided 28 AKI samples and 19 control samples. The data were conducted the standardized processing of log2-transformation for further analysis. Data normalization was performed using the R software package.

### Recognition of COVID-19 and AKI related DEGs

For exploring the shared gene signatures between COVID-19 and AKI, the assessment of differential gene expression was first carried out between disease samples and control samples via “Limma” package [18]. The “adjusted *P* < 0.05 and Fold Change > 2” were determined as the filter criteria for the differential expression of mRNAs. Subsequently, volcano plot and heatmap were employed to exhibit the screening DEGs in COVID-19 and AKI cohorts, respectively. Finally, the common DEGs between COVID-19 and AKI cohorts were determined by the intersection using Venn diagram [19].

### Function enrichment analysis

The GO and KEGG analyses were carried out utilizing the “ggplot2” and “Cluster Profiler” packages. Moreover, GO analysis, such as the biological process (BP), cellular component (CC), and molecular function (MF), of the common DEGs between COVID-19 and AKI cohorts were also conducted using the same method. To further confirm the potential function of the differential expression genes, the data was analyzed through functional enrichment method. For the enrichment results, FDR < 0.05 was considered to be enriched to the meaningful pathway.

### Identification of the key gene signatures between COVID-19 and AKI

LASSO regression algorithm can be utilized to establish the optimal model based on the selected genes for disease diagnosis and the prediction of prognosis [20]. LASSO algorithm is the most common method for the selection of key gene signatures, and 10-fold cross-validation was used. The key gene signatures between COVID-19 and AKI were obtained from the common DEGs using LASSO method via the “glmnet” package [21]. The coefficients of selected features are shown by lambda parameter. Additionally, receiver operating characteristic (ROC) analysis was conducted to evaluate the predictive ability of these key gene signatures and the model by calculating the area under the curve (AUC) value [22].

### Subgroup analysis in COVID-19 based on the key gene signatures

Owing to individual differences in different patients with COVID-19, we further carried out the subgroup analysis in COVID-19 patients based on the key gene signatures using the consensus clustering analysis [16]. The consistency analysis was conducted by using the “ConsensusClusterPlus” R package. According to the relative change of the area under the cumulative distribution function (CDF) curve, the optimal k value can be determined. The COVID-19 patients were ultimately classified into two different subgroups (C1 and C2). Subsequently, we identified the DEGs between C1 and C2 subgroups, and then the function analysis of DEGs was conducted.

### Protein–protein interaction (PPI) network analysis

The interactive proteins to the key gene signatures were further explored via PPI network analysis. As the shared gene signatures were input in the NetworkAnalyst 3.0 platform, the PPI network was created by selecting “STRING Interactome” database and setting the parameters “the confidence score cutoff (900)”. Then, the network was needed to be visualized using Cytoscape (Version: 3.8.0).

### Regulatory networks analysis of gene signature, transcription factor (TF), and miRNA

The systematic regulatory networks analysis, including TF-gene interaction network, gene-miRNA interaction network, and TF-miRNA coregulatory network, were conducted via NetworkAnalyst 3.0 platform (https://www.networkanalyst.ca). Specifically, we selected the ENCODE ChIP-seq database for TF-gene interaction network analysis. For gene-miRNA interaction network, we selected the miRTarBase v8.0 database, containing comprehensive experimentally validated miRNA-gene interaction data. Finally, we constructed TF-miRNA coregulatory network via the RegNetwork repository.

### Statistics analysis

All R packages mentioned in this study were performed via R v4.0.3 software. The statistical *P* value less than 0.05 was acknowledged statistically significant.

## Results

### Recognition of the shared DEGs between COVID-19 and AKI

**Fig. 1:**
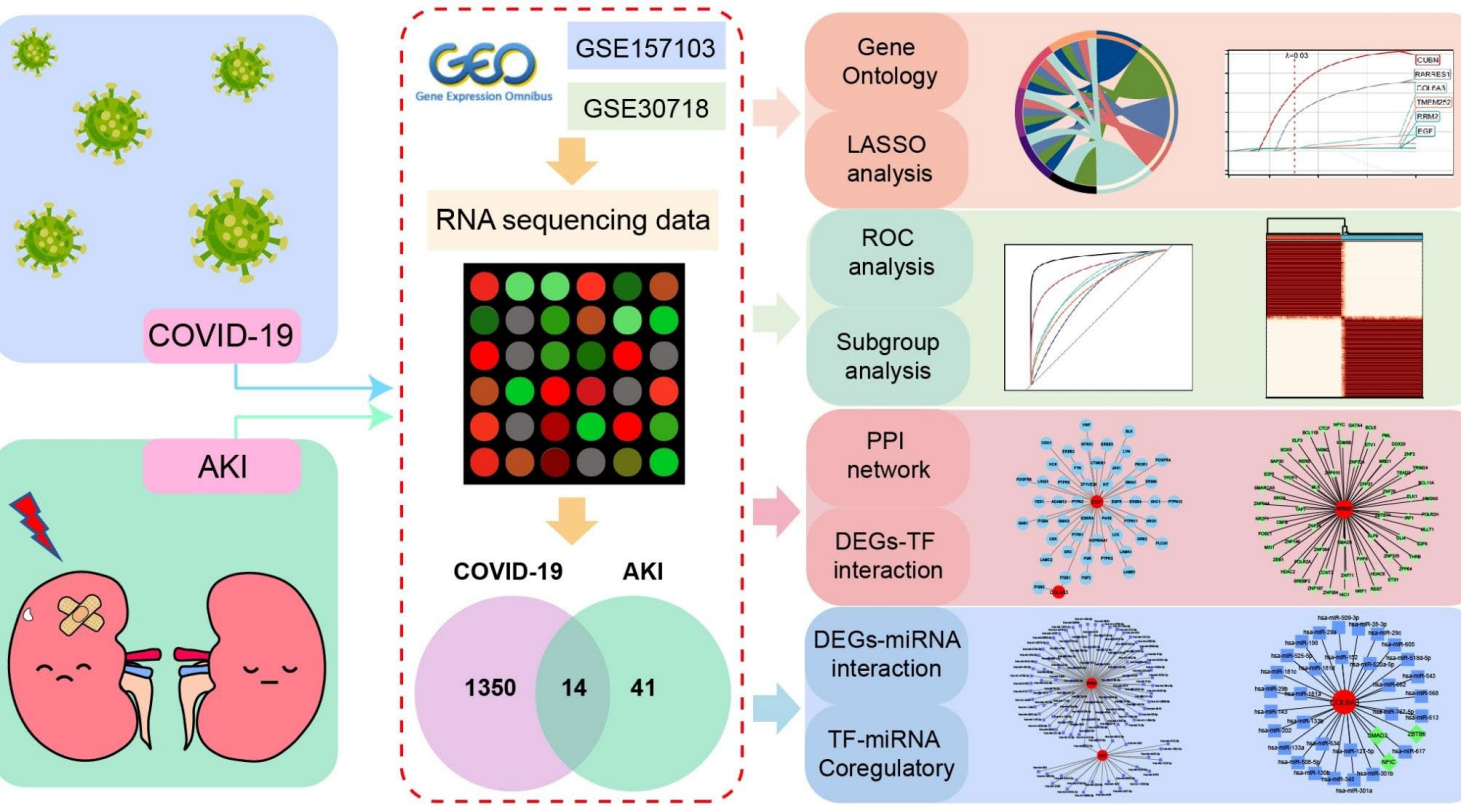
Flow diagram displaying the overall study protocol. Through differential gene expression analysis of GSE157103 dataset in COVID-19 and GSE30718 dataset in acute kidney injury (AKI), the differentially expressed genes (DEGs) were identified and then subsequent analyses including function enrichment, LASSO, ROC, subgroup analysis, PPI network, and gene regulatory network were performed

**Fig. 2:**
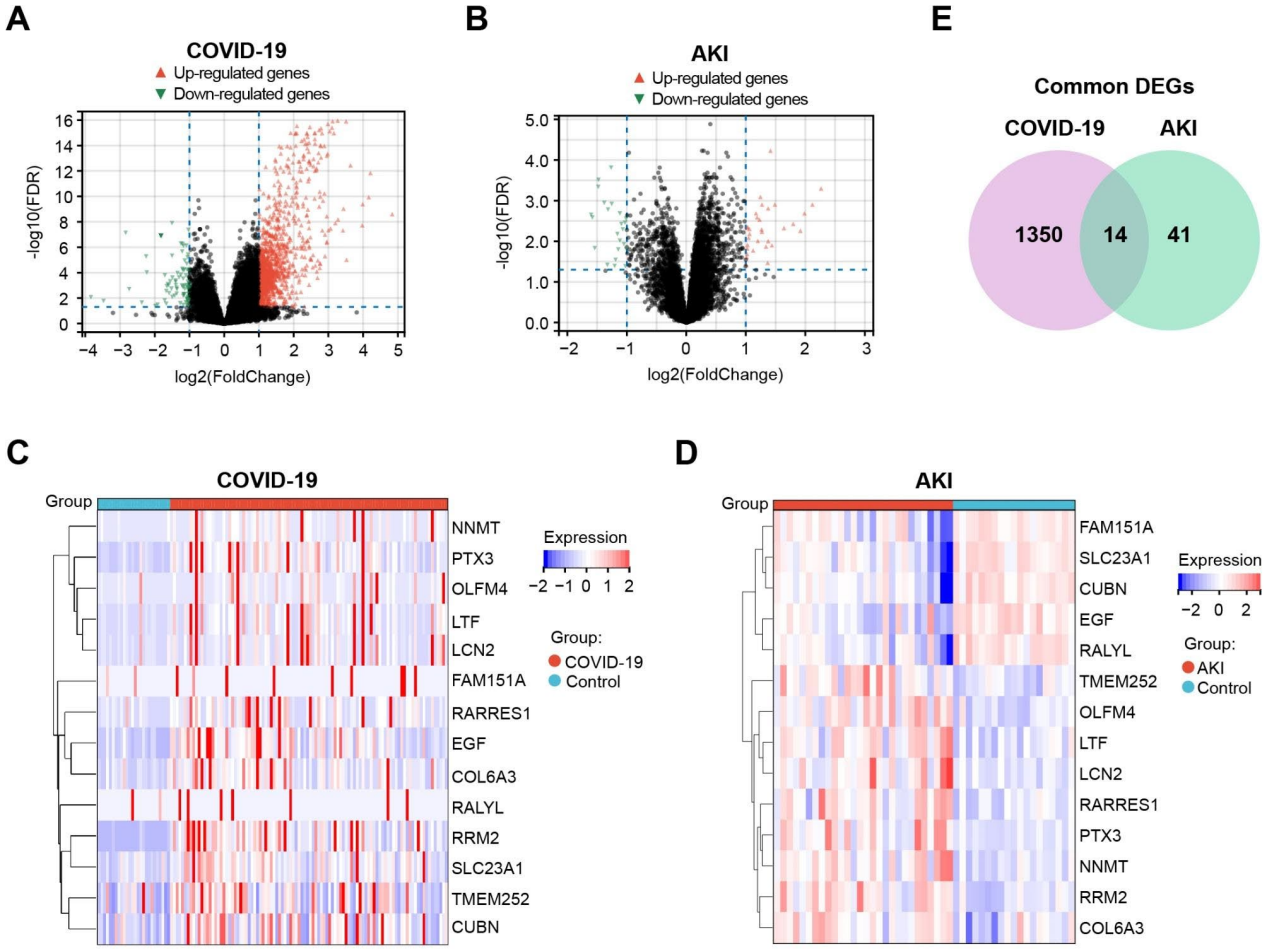
Identification of the common differentially expressed genes (DEGs) between COVID-19 and acute kidney injury (AKI). (**A, B**) Volcano plots displaying DEGs of (**A**) COVID-19 and (**B**) AKI. Red triangle denotes up-regulated gene, and green triangle denotes down-regulated gene. (**C, D**) Heatmap displaying the DEGs expression (adjusted P < 0.05 and Fold Change > 2) of (**C**) COVID-19 and (**D**) AKI. (**E**) Venn diagram displaying 14 common DEGs from the intersection between COVID-19 and AKI

**Fig. 3:**
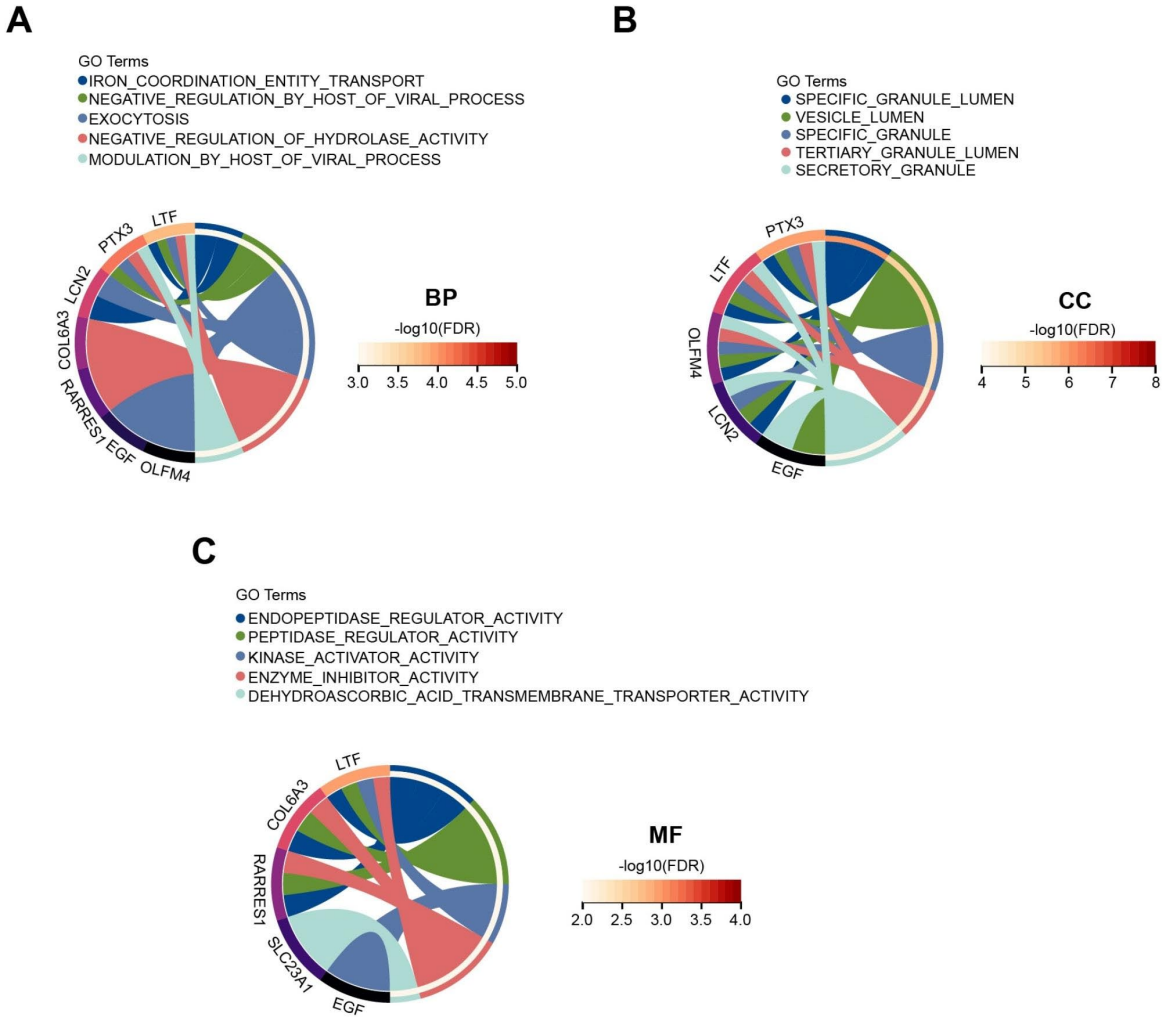
Gene ontology (GO) analysis of 14 common DEGs. (**A-C**) Circle graph displaying the top5 pathway of (**A**) BP (biological process), (**B**) CC (cellular component), and (**C**) MF (molecular function) in GO analysis

The overall study strategy was displayed through a flow diagram (Fig. 1). In order to identify the relevance between COVID-19 and AKI in terms of gene expression, we analyzed the COVID-19 RNA-seq data using GSE157103 dataset and AKI microarray data using GSE30718 dataset. Through differential gene expression analysis with the filter criteria “adjusted P < 0.05 and Fold Change > 2”, we identified 1364 DEGs (1259 up-regulation and 105 down-regulation) in COVID-19 dataset (Fig. 2A) and 55 DEGs (32 up-regulation and 23 down-regulation) in AKI dataset (Fig. 2B). The detail information of DEGs in both COVID-19 cohort and AKI cohort could be acquired from Supplementary Material-Data. Meanwhile, hierarchical clustering analysis was employed to display the expression of DEGs by heatmap (Fig. 2C and D). We further conducted the intersection analysis using Venn, and 14 common DEGs (RRM2, OLFM4, LTF, EGF, PTX3, NNMT, SLC23A1, LCN2, FAM151A, RALYL, TMEM252, RARRES1, COL6A3, CUBN) were ultimately determined from COVID-19 and AKI datasets (Fig. 2E). Finally, GO enrichment analysis of the 14 common DEGs were conducted, and the Top5 pathway of BP, CC, and MF were exhibited via circle graph (Fig. 3A–C).

### Identification of the key gene signatures between COVID-19 and AKI

**Fig. 4:**
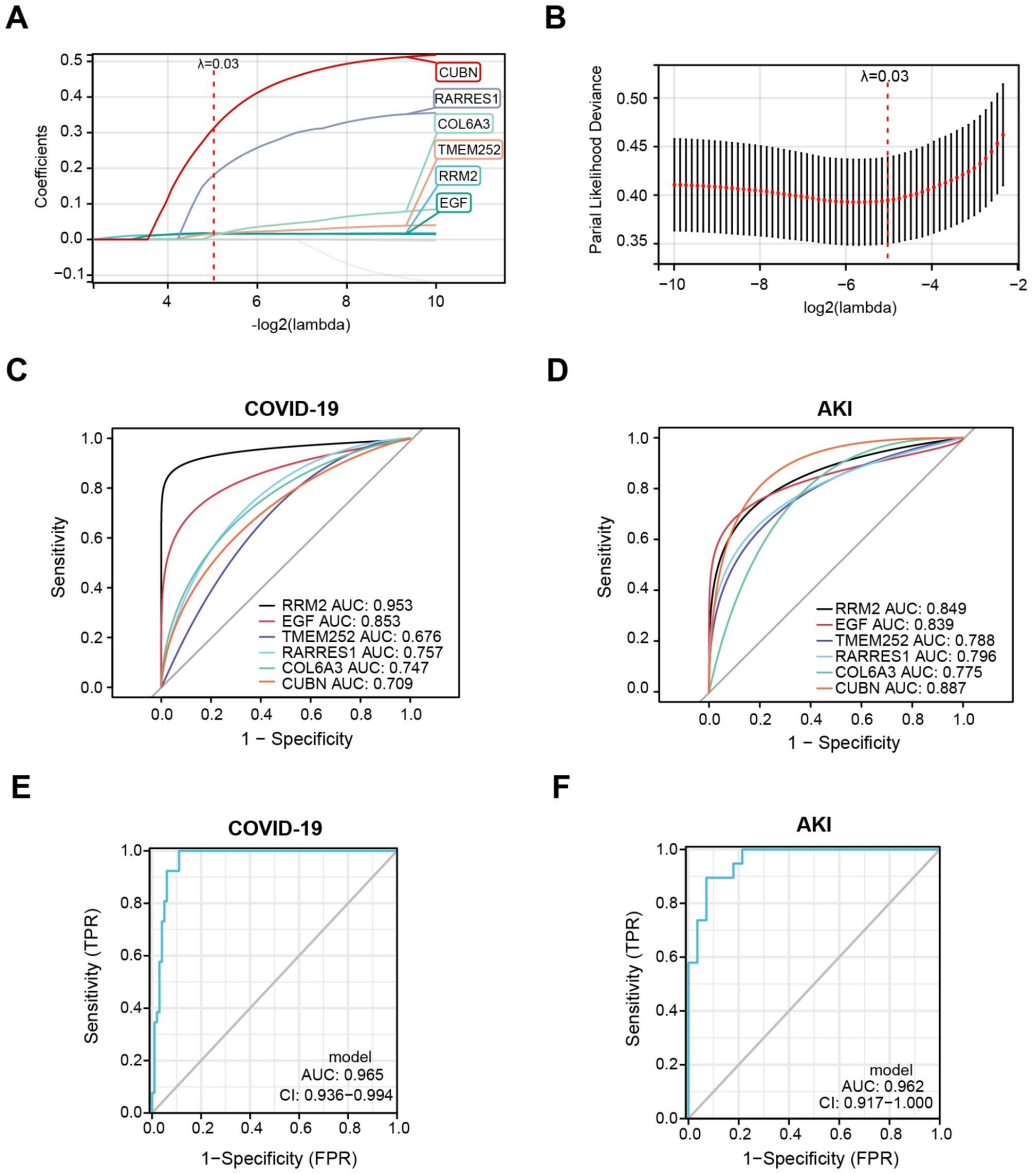
Identification of the shared gene signatures between COVID-19 and AKI. (**A, B**) Screening of the shared gene signatures from the 14 common DEGs of COVID-19 and AKI using the LASSO algorithm. A total of 6 candidate gene signatures (RRM2, EGF, TMEM252, RARRES1, COL6A3, CUBN) were determined. (**C, D**) Receiver operating characteristic (ROC) curve displaying the prediction efficiency of the 6 gene signatures in (**C**) COVID-19 and (**D**) AKI. (**E, F**) ROC curve displaying the prediction efficiency of the model in (**E**) COVID-19 and (**F**) AKI

Based on 14 common DEGs, the LASSO algorithm was applied to determine the key gene signatures between COVID-19 and AKI. The results revealed that 6 key gene signatures (RRM2, EGF, TMEM252, RARRES1, COL6A3, CUBN) were recognized to have great influences on COVID-19 and AKI (Fig. 4A, B). Based on the coefficients of 6 gene signatures, the model score was determined by summing of each gene multiplying with the corresponding coefficient. The calculation formula was as follows: Model Score = 0.015*RRM2 + 0.017*EGF + 0.016*TMEM252 + 0.181*RARRES1 + 0.011*COL6A3 + 0.313*CUBN. Then, we evaluated the diagnostic efficiency of these key gene signatures, and ROC analysis revealed that these key gene signatures had certain predictive value in COVID-19 (RRM2 (AUC: 0.953), EGF (AUC: 0.853), TMEM252 (AUC: 0.676), RARRES1 (AUC: 0.757), COL6A3 (AUC: 0.747), CUBN (AUC: 0.709), Fig. 4C) and AKI (RRM2 (AUC: 0.849), EGF (AUC: 0.839), TMEM252 (AUC: 0.788), RARRES1 (AUC: 0.796), COL6A3 (AUC: 0.775), CUBN (AUC: 0.887), Fig. 4D), respectively. Notably, the model constructed by 6 key gene signatures presented a considerable diagnostic efficiency in both COVID-19 (AUC: 0.965, Fig. 4E) and AKI (AUC: 0.962, Fig. 4F).

### Identification of key gene signatures-associated subgroups in COVID-19 cohort

Owing to individual differences in COVID-19 patients, we further performed the subgroups analysis in COVID-19 cohort based on these key gene signatures utilizing the consensus clustering tool. Then, 100 COVID-19 samples were divided into two distinct subgroups, including cluster 1 (C1, n = 48) and cluster 2 (C2, n = 52) (Figure S1 A-C, Supplementary Material). We further identified 441 DEGs (430 up-regulation and 11 down-regulation) between C1 and C2 subgroups (Figure S1 D, Supplementary Material). The detail information of DEGs between C1 and C2 subgroups could be acquired from Supplementary Material-Data. Moreover, hierarchical clustering analysis was employed to display the expression of DEGs by heatmap (Figure S1 E, Supplementary Material). Finally, enrichment analyses indicated that the GO analysis of DEGs mainly concentrated in organelle fission, nuclear division, chromosomal region, ATPase activity, and so on (Figure S1 F, Supplementary Material). The KEGG analysis [23] of DEGs mainly concentrated in cell cycle, oocyte meiosis, cellular senescence, and so on (Figure S1 G, Supplementary Material).

### Identification of protein–protein interaction (PPI) network

**Fig. 5:**
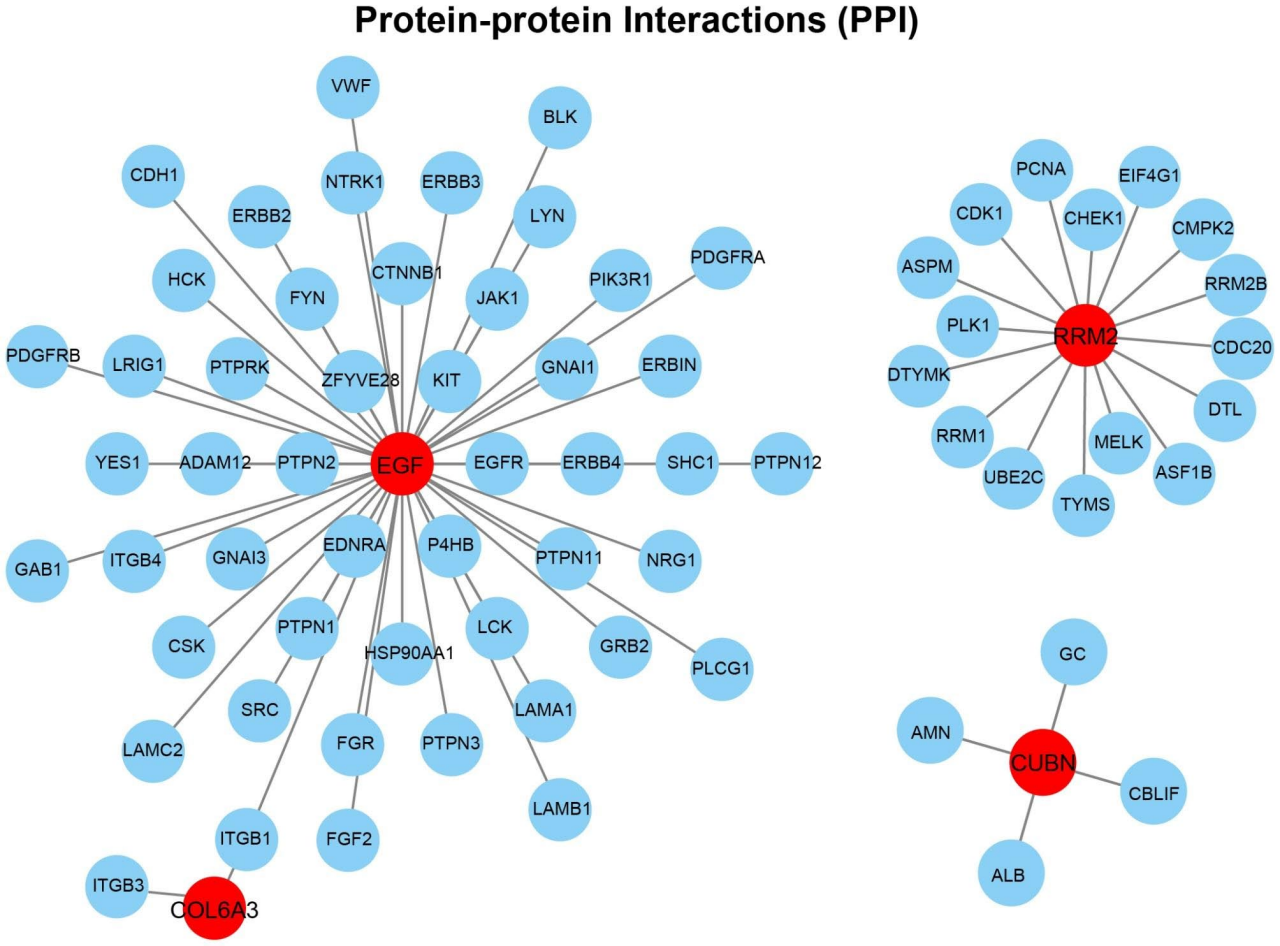
Protein-protein interaction (PPI) network of the key gene signatures. Red circles represent the proteins corresponding to the candidate gene signatures, and blue circles represent the interrelated proteins

We constructed PPI network using the STRING interactome [24] model in NetworkAnalyst 3.0 platform. As shown in Fig. 5, there presented three PPI networks, of which EGF-COL6A3 network contained 51 nodes and 50 edges, RRM2 network contained 17 nodes and 16 edges, and CUBN network contained 5 nodes and 4 edges. The detail information of nodes in PPI network could be acquired from Table S1 (Supplementary Material).

### Regulatory networks analysis of gene signature, transcription factor (TF), and miRNA

**Fig. 6:**
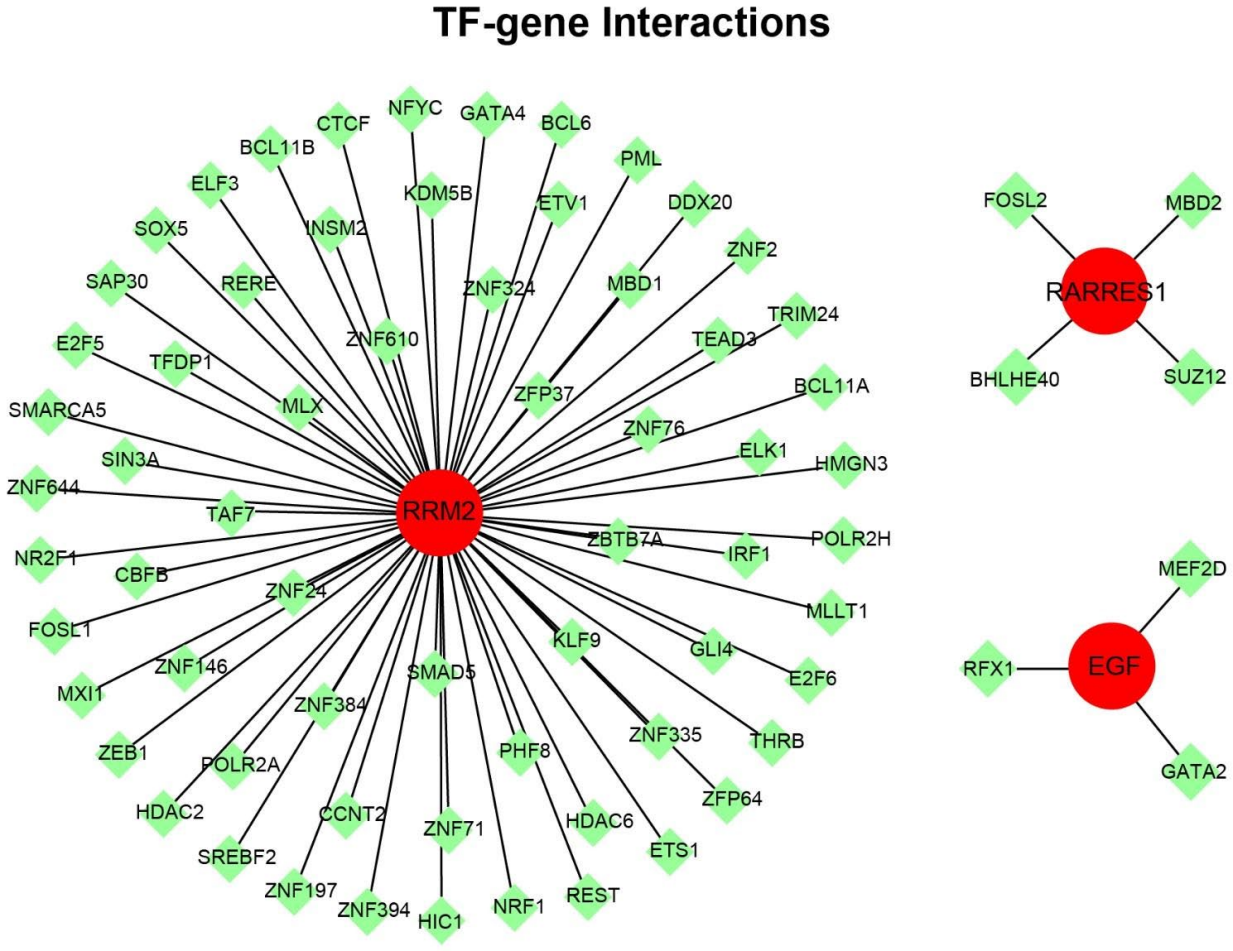
The construction of transcription factor (TF)-gene interaction network. Red circles represent the candidate gene signatures, and green rhombuses represent the interrelated transcription factors to gene signatures

**Fig. 7:**
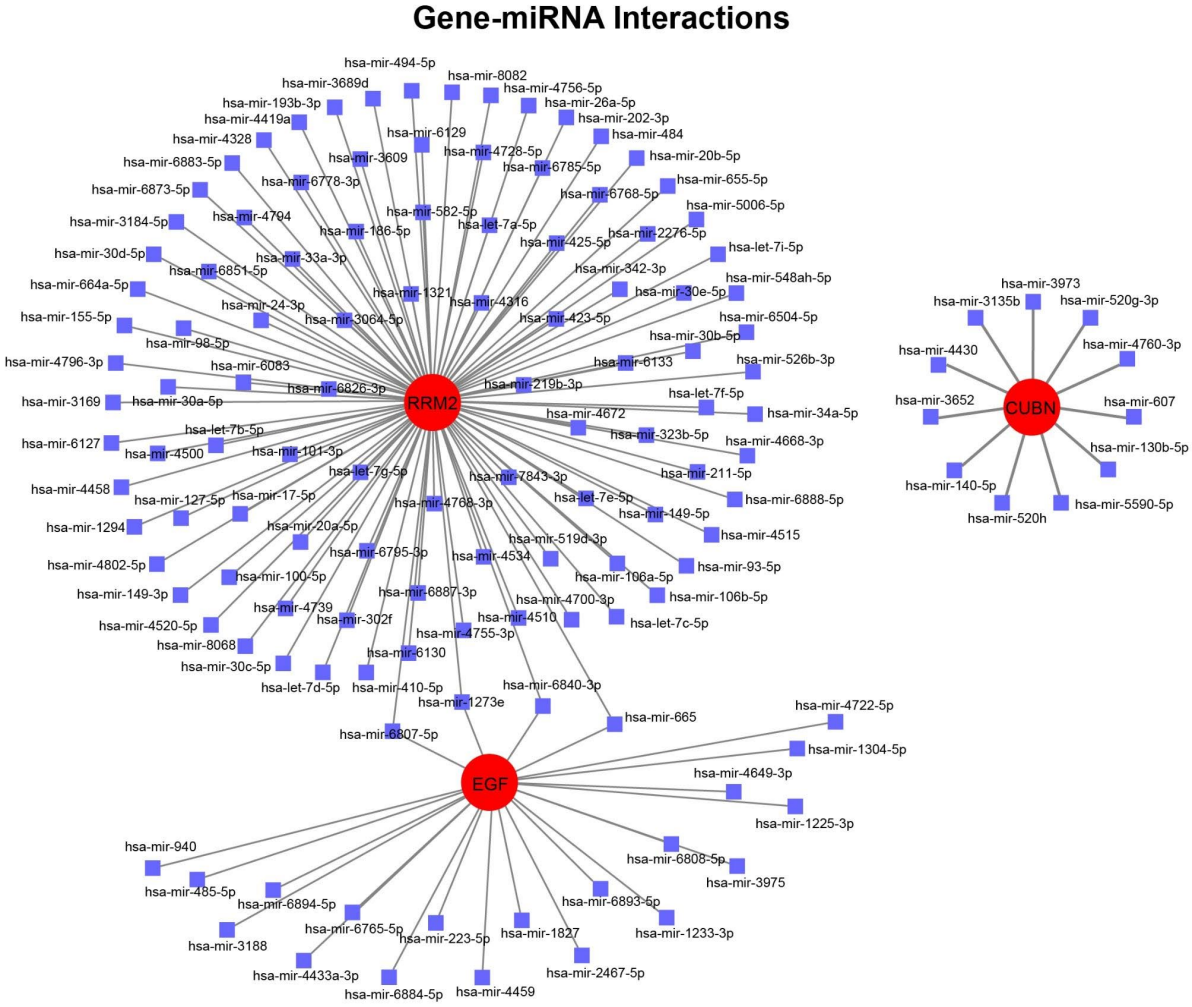
The construction of gene-miRNA interaction network. Red circles represent the candidate gene signatures, and blue squares represent the interrelated miRNA to gene signatures

We first analyzed the association between gene signature and transcription factor using NetworkAnalyst 3.0 platform. As shown in Fig. 6, there presented three TF–gene interaction networks, of which RRM2 was regulated by 64 TF genes, RARRES1 was regulated by 4 TF genes, and EGF was regulated by 3 TF genes. The detail information of gene signatures and TF genes in TF–gene interaction networks could be acquired from Table S2 (Supplementary Material). These results demonstrated that there had high interaction between TF genes and the key gene signatures. Meanwhile, the regulatory network of gene signature and miRNA was investigated. As shown in Fig. 7, two gene-miRNA networks were constructed, of which RRM2-EGF-miRNA network included 122 nodes (2 gene signatures and 120 miRNAs) and 124 edges, CUBN-miRNA network included 12 nodes (1 gene signature and 11 miRNAs) and 11 edges. The detail information of gene signatures and miRNAs in gene-miRNA interaction network could be acquired from Table S3 (Supplementary Material). These results suggested there had the complex regulatory networks between gene signatures and miRNAs. Finally, we carried out TF-miRNA coregulatory network analysis to explore the correlation of miRNA and TF with the key gene signatures using NetworkAnalyst 3.0 platform. As shown in Figure S2 (Supplementary Material), two TF-miRNA coregulatory networks were constructed, of which COL6A3 network included 35 nodes (3 TF genes and 31 miRNAs) and 34 edges, and RRM2-EGF-RARRES1 network included 34 nodes (23 TF genes and 8 miRNAs) and 33 edges. The detail information of gene signatures, TF genes and miRNAs in TF-miRNA coregulatory network could be acquired from Table S4 (Supplementary Material). These results showed that gene signatures, TF genes and miRNAs formed the complex gene expression regulatory network.

## Discussion

Many studies have found that spike protein of SARS-CoV-2 can specifically act on angiotensin-converting enzyme 2 (ACE2) receptor, leading to COVID-19 infection [25, 26]. According to research findings, SARS-CoV-2 infection can directly result in cardiovascular and renal disease owing to the pathogenic mechanism of SARS-CoV-2 interacting with ACE2 protein [27]. In fact, ACE2 is widely expressed in multiple organs, including kidney, and COVID-19 affects human kidney organoids by direct invasion [28, 29]. Moreover, an analysis in 85 COVID-19 patients reveals that ACE2 receptor is expressed on human renal tubules and SARS-CoV-2 directly participates in the infection of human renal tubules [30]. According to the examination of kidney tissues in died COVID-19 patients, the virus presents a certain propensity to renal invasion and induces its damage, which is closely related to high AKI incidence and poor prognosis [31]. Based on the current studies, AKI appears to increase the incidence of severe and death in patients with COVID-19.

On the one hand, SARS-CoV-2 can directly act on renal cells inducing cells injury accompanied by subsequent fibrosis. On the other hand, the immune system recognizes viral particles and initiates an inflammatory response, causing the damage of healthy tissues. Therefore, one of the most important issues that researchers must urgently address is whether there is some way to recognize and decrease the incidence of AKI in COVID-19 patients. Many rigorous and diverse studies have been conducted for exploring meaningful and broadly applicable data. A recent study of COVID-19 patients in African reveals that there presents the correlation of APOL1 kidney risk variants with high AKI incidence [32]. From a multi-center study in COVID-19 patients, cytokine storm and secondary bacterial infections perform a vital role in the development of AKI [33]. Therefore, exploration of the potential molecular pathogenesis between AKI and COVID-19 has become extremely important. The shared gene signatures and gene regulatory network between COVID-19 and AKI has not been well understood. The investigations of gene signatures and regulatory network contribute to revealing the potential mechanism of the development of AKI in COVID-19 patients.

Our study focused on exploring the shared gene signatures and gene regulatory network between COVID-19 and AKI from gene expression and regulation aspects. We first screened out the DEGs in COVID-19 and AKI cohorts, respectively. Through taking the intersection, the shared DEGs between COVID-19 and AKI were obtained. Then, the most representative gene signatures were ultimately determined by machine learning method. The model constructed by these key gene signatures had a high predictive efficacy in both COVID-19 and AKI cohorts. The results suggested that these gene signatures could be considered as the key shared genes in the pathogenesis between COVID-19 and AKI. These candidate gene signatures may contribute to predicting the risk of AKI, understanding of the pathogenesis of renal injury, and guiding the pertinent treatment in COVID-19 patients.

Based on these key gene signatures, two different subgroups in COVID-19 cohort were identified, and the DEGs between the two subgroups concentrated in cell division and cell cycle. These results suggested that the COVID-19 patients exhibited individual differences. In addition, according to these gene signatures, we constructed the comprehensive gene regulatory networks, including PPI, transcription factor (TF)-gene interaction, gene-miRNA interaction, and TF-miRNA coregulatory networks. Through the system biology analyses, involved in transcription factor, miRNA, target gene, and protein, the comprehensive gene regulatory networks were created, contributing to uncovering the internal connection of the expression regulation of these key gene signatures.

This study had some limitations. Despite many samples of COVID-19 and AKI had been collected in this study, the sample size was still inadequate. Thus, it is necessary to collect enough samples for further validation. Moreover, the significant value of these gene signatures for predicting clinical outcomes and offering new therapeutic targets in COVID-19 patients are needed to be explored further. We hope that adequate clinical evidence will be obtained to support this finding in future research.

## Conclusions

In conclusion, this study explored and determined the share gene signatures between COVID-19 and AKI, and constructed the comprehensive gene regulatory network, which contributed to better identifying the common pathogenesis and providing the potential therapeutic targets for clinical management for COVID-19 and AKI.

### Electronic supplementary material

Below is the link to the electronic supplementary material.

Supplementary Material 1

Supplementary Material 2

Supplementary Material 3

Supplementary Material 4

Supplementary Material 5

Supplementary Material 6

Supplementary Material 7

## Data Availability

All data and materials of this study can be obtained from the corresponding author under reasonable request.

## Abbreviations

ACE2: Angiotensin-converting enzyme 2
AKI: Acute kidney injury
ARDS: Acute respiratory distress syndrome
AUS: Area under the curve
BP: Biological process
CC: Cellular component
COVID-19: Coronavirus disease 2019
DEGs: Differentially expressed genes
GEO: Gene expression omnibus
GO: Gene ontology
KEGG: Kyoto encyclopedia of genes and genomes
LASSO: Least absolute shrinkage and selection operator
MF: Molecular function
PPI: Protein?protein interaction
ROC: Receiver operating characteristic
RRT: Renal replacement therapy
SARS-CoV-2: Severe acute respiratory syndrome coronavirus 2
TF: Transcription factor
WHO: World health organization
