# Supplementary material for: Identification of the potential association between SARS-CoV-2 infection and acute kidney injury based on the shared gene signatures and regulatory network
Source: BMC Infect Dis. 2023 Oct 3;23:655. doi: 10.1186/s12879-023-08638-6 (PMC10548629; doi:10.1186/s12879-023-08638-6)
Supplement: Supplementary file 1 — Supplementary Material 1 [file 12879_2023_8638_MOESM1_ESM.pdf]

Supplementary Material

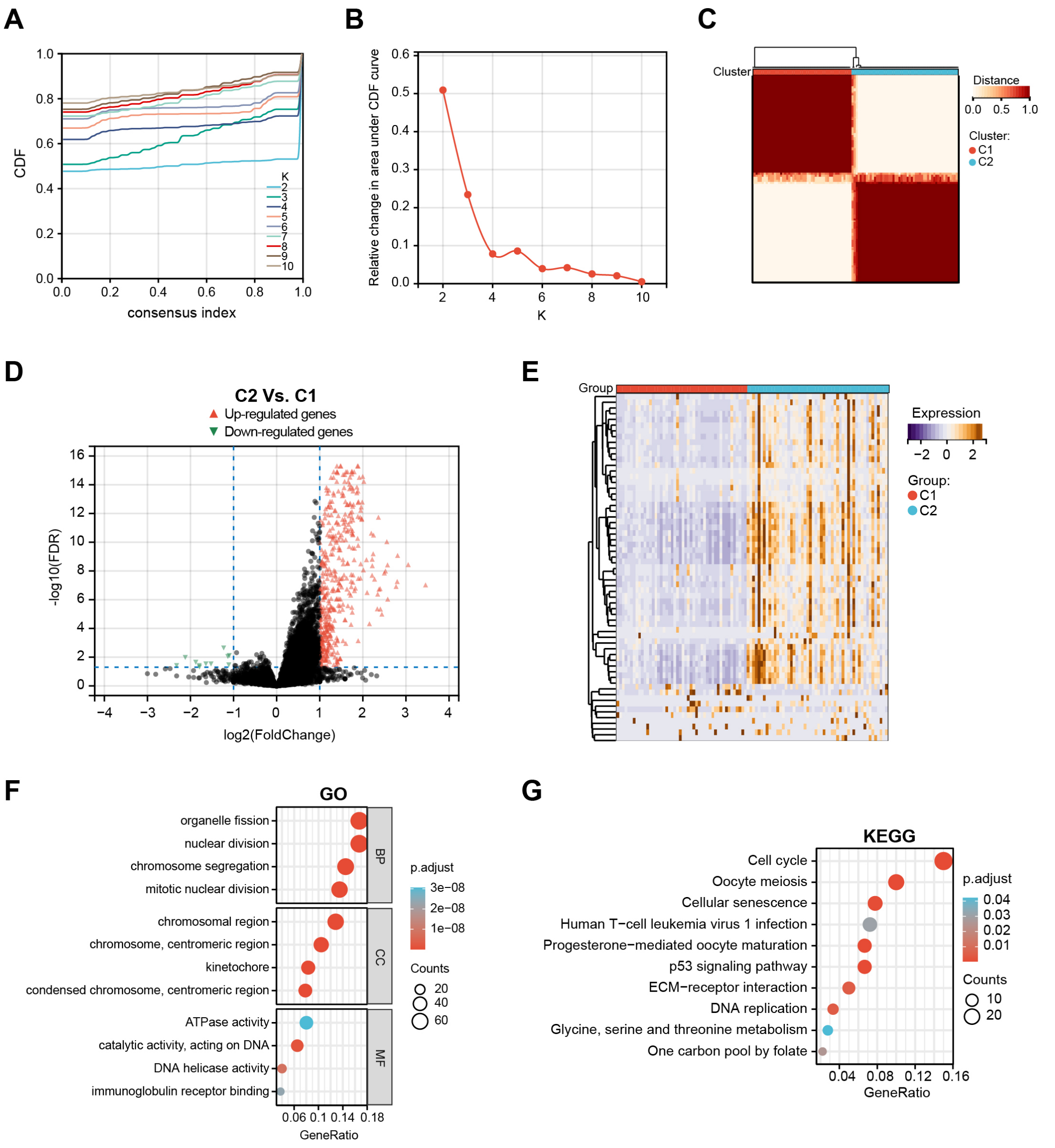

Figure S1. Identification of the key gene signatures-related subgroups in COVID-19 cohort by consensus clustering analysis. (A) Consensus clustering of the cumulative distribution function (CDF) curve for k = 2 to 10. (B) Elbow plot depicting relative change in area under CDF curve. (C) Heatmap displaying the consensus clustering (k = 2). COVID-19 samples were classified into two different subgroups (C1 and C2). (D) Volcano plots displaying the DEGs

(adjusted $P < 0.05$ and Fold Change > 2). Red triangle denotes up-regulated gene, and green triangle denotes down-regulated gene. (E) Heatmap displaying the DEGs expression in the two subgroups. (F, G) Bubble diagram displaying (F) GO and (G) KEGG function enrichment analysis of these DEGs in the two subgroups.
