# Supplementary figures and images for: Identification of the potential association between SARS-CoV-2 infection and acute kidney injury based on the shared gene signatures and regulatory network
Source: BMC Infect Dis. 2023 Oct 3;23:655. doi: 10.1186/s12879-023-08638-6 (PMC10548629; doi:10.1186/s12879-023-08638-6)

## TF-miRNA Coregulatory Network

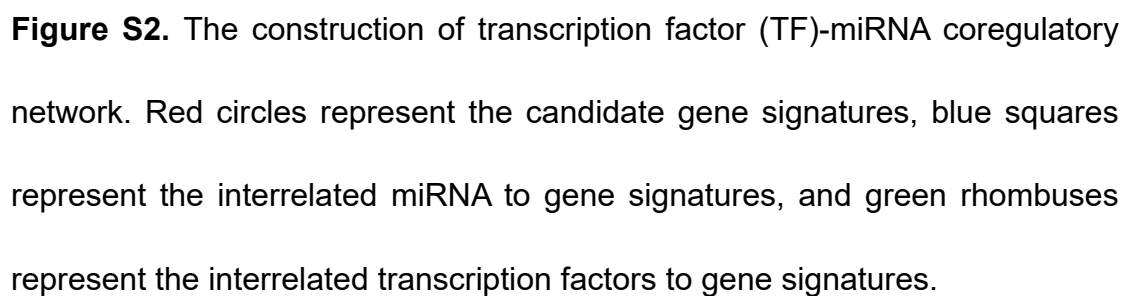

Supplement: Supplementary file 2 — Supplementary Material 2 [file 12879_2023_8638_MOESM2_ESM.pdf]
