# Supplementary material for: Identification of the potential association between SARS-CoV-2 infection and acute kidney injury based on the shared gene signatures and regulatory network
Source: BMC Infect Dis. 2023 Oct 3;23:655. doi: 10.1186/s12879-023-08638-6 (PMC10548629; doi:10.1186/s12879-023-08638-6)
Supplement: Supplementary file 3 — Supplementary Material 3 [file 12879_2023_8638_MOESM3_ESM.pdf]

Supplementary Data

COVID-19-DEGs		AKI-DEGs		C1-C2 subgroups DEGs	
IGHG1	Up	UGT1A6	Up	IGHG1	Up
IFI27	Up	SERPINA3	Up	JCHAIN	Up
GLDC	Up	RRM2	Up	GLDC	Up
JCHAIN	Up	FCGR3A	Up	IGHG3	Up
IGHG3	Up	OLFM4	Up	IGLC2	Up
SDC1	Up	TIMP1	Up	IGKC	Up
TNFRSF17	Up	LY96	Up	MZB1	Up
OTOF	Up	CD163	Up	IGF1	Up
PBK	Up	ITGB6	Up	SDC1	Up
BHLHA15	Up	NNMT	Up	BHLHA15	Up
MCM10	Up	C2CD4A	Up	IGHA1	Up
IGLC2	Up	SLPI	Up	IGHG2	Up
IGLC1	Up	VSIG4	Up	IGLC1	Up
TOP2A	Up	C1S	Up	IGLL5	Up
DTL	Up	PTX3	Up	TXNDC5	Up
IGLC3	Up	QPCT	Up	IGLC3	Up
BUB1B	Up	COL3A1	Up	IGHG4	Up
HMMR	Up	LTF	Up	DERL3	Up
CDCA2	Up	C1QB	Up	CAV1	Up
TYMS	Up	PLTP	Up	GPRC5D	Up
MZB1	Up	TMEM252	Up	TNFRSF17	Up
MELK	Up	RARRES1	Up	UCHL1	Up
BUB1	Up	LCN2	Up	MET	Up
IGLL5	Up	S100A2	Up	PYCR1	Up
DEPDC1B	Up	COL6A3	Up	IGHM	Up
DIAPH3	Up	VCAN	Up	PLAAT2	Up
CDC25A	Up	C3	Up	SLC16A14	Up
KIF20A	Up	ADAMTS1	Up	BUB1	Up
GPRC5D	Up	EVI2A	Up	MIXL1	Up
H2BC17	Up	COL1A1	Up	CDCA2	Up
E2F8	Up	AKR1B10	Up	BUB1B	Up
UCHL1	Up	MMP7	Up	PBK	Up
ABCA13	Up	SLC23A3	Down	E2F8	Up
IFI44L	Up	SOST	Down	HASPIN	Up
TXNDC5	Up	SLC34A1	Down	ANLN	Up
DLGAP5	Up	KLK1	Down	DLGAP5	Up
CDC45	Up	MRO	Down	TSHR	Up
CAV1	Up	PVALB	Down	TOP2A	Up
CDK1	Up	TMEM207	Down	DTL	Up
RRM2	Up	RNF212B	Down	SKA3	Up
IGF1	Up	EGF	Down	KCNN3	Up
ASPM	Up	LEAP2	Down	E2F7	Up
EXO1	Up	SLC12A3	Down	CDC25A	Up
DEPDC1	Up	RALYL	Down	IGLC7	Up
SKA3	Up	CUBN	Down	TTK	Up
KIF15	Up	SLC23A1	Down	MCM10	Up
EPSTI1	Up	KNG1	Down	POLQ	Up
RSAD2	Up	RDH12	Down	ITM2C	Up
IGHA1	Up	CTXN3	Down	RRM2	Up
ANKRD45	Up	HAO2	Down	MELK	Up
KCNN3	Up	ETNPPL	Down	KIF15	Up
E2F7	Up	GPC5	Down	B9D1	Up
MKI67	Up	FAM151A	Down	PARM1	Up
CENPA	Up	AFM	Down	KIF20A	Up

POLQ	Up
IGKC	Up
NCAPG	Up
OLFM4	Up
MMP8	Up
TTK	Up
KIF4A	Up
CDC6	Up
IGHG2	Up
GINS1	Up
DSCC1	Up
KIF14	Up
HJURP	Up
CCNE2	Up
ESCO2	Up
MYBL2	Up
TICRR	Up
CEACAM8	Up
IGHG4	Up
INHBA	Up
OAS3	Up
CEP55	Up
KLHL14	Up
NDUFC2-KCTD14	Up
GTSE1	Up
KIF18A	Up
RAD54L	Up
PLAAT2	Up
DOC2B	Up
PYCR1	Up
DEFA3	Up
TRIP13	Up
CDC20	Up
STIL	Up
ANLN	Up
TPX2	Up
CENPF	Up
FAM111B	Up
ERG	Up
CCNB2	Up
SLC16A14	Up
CDCA5	Up
ZWINT	Up
CKAP2L	Up
ARHGAP42	Up
MND1	Up
SMTNL1	Up
BIRC5	Up
BHLHE41	Up
USP18	Up
RPH3A	Up
TROAP	Up
MIXL1	Up
RAD51	Up
ZBED6	Up
CMPK2	Up
IFIT1	Up
CLDN24	Up

MIOX	Down

EXO1	Up
ARHGAP42	Up
ASPM	Up
ALDH1L2	Up
DEPDC1B	Up
POU2AF1	Up
MYBL2	Up
KIF4A	Up
CENPF	Up
HJURP	Up
CENPA	Up
KIF14	Up
NEIL3	Up
KLHL14	Up
CDCA3	Up
HMMR	Up
TYMS	Up
SHCBP1	Up
CDC20	Up
TNFRSF13B	Up
TRIP13	Up
ABCB9	Up
HPDL	Up
CDK1	Up
OXCT2	Up
IGHA2	Up
DEPDC1	Up
CHPF	Up
MKI67	Up
CEP55	Up
KIFC1	Up
NCAPH	Up
TPX2	Up
CDCA5	Up
NCAPG	Up
ZWINT	Up
COL4A4	Up
MYO1D	Up
TICRR	Up
STIL	Up
CDC45	Up
DIAPH3	Up
RAPGEF5	Up
NUGGC	Up
FAM111B	Up
KIF2C	Up
KIF11	Up
BIRC5	Up
CCNE2	Up
NEK2	Up
CADM1	Up
HELLS	Up
ABCA13	Up
CEP128	Up
RAD54L	Up
GINS1	Up
IQGAP3	Up
RAD51AP1	Up

KIF11	Up
NEK2	Up
LOC389831	Up
SHCBP1	Up
KNL1	Up
HASPIN	Up
NEIL3	Up
PARM1	Up
IQGAP3	Up
PHKA1	Up
LTF	Up
OLR1	Up
CCNA1	Up
KIF2C	Up
ORC1	Up
TARM1	Up
H4C8	Up
PCSK9	Up
H2BC7	Up
CCNA2	Up
CENPE	Up
KIFC1	Up
ALDH1L2	Up
GINS2	Up
DEFA4	Up
NCAPH	Up
MOXD1	Up
CTSG	Up
OASL	Up
PLK4	Up
APOBEC3B	Up
PALM2AKAP2	Up
CENPI	Up
CEACAM6	Up
ZFY	Up
CIT	Up
CCNB1	Up
NUSAP1	Up
CDCA3	Up
SUCNR1	Up
EME1	Up
HELLS	Up
SPINK4	Up
ESPL1	Up
MET	Up
FBXO43	Up
POLE2	Up
MATN2	Up
CD38	Up
CDC25C	Up
TNFRSF13B	Up
BIVM-ERCC5	Up
CHEK1	Up
DERL3	Up
FAP	Up
SLC2A5	Up
CENPK	Up
POSTN	Up

CENPE	Up
LAX1	Up
CDC6	Up
ESPL1	Up
PLK4	Up
ESCO2	Up
RAD51	Up
SLC2A5	Up
KNL1	Up
CCNB2	Up
LMAN1	Up
ORC1	Up
UGT2B17	Up
MOXD1	Up
CENPI	Up
KIF18A	Up
WDR62	Up
BHLHE41	Up
TROAP	Up
DSCC1	Up
NUF2	Up
POGLUT2	Up
CKAP2L	Up
CDT1	Up
POLE2	Up
SPAG5	Up
BVES	Up
MMP8	Up
DENND5B	Up
CTSG	Up
CIT	Up
ERCC6L	Up
PALD1	Up
DOK5	Up
CNKSR1	Up
IRF4	Up
TPD52	Up
CIP2A	Up
CHAC2	Up
TK1	Up
CENPM	Up
LOC283710	Up
TENT5C	Up
EME1	Up
FAM83D	Up
COBLL1	Up
FOXM1	Up
GINS2	Up
ZBTB32	Up
PERP	Up
KIR2DS1	Up
CRYBB1	Up
CEACAM8	Up
CCNA2	Up
CRISP2	Up
ERG	Up
TBX1	Up
DEFA4	Up

TMEM52B	Up
FOXM1	Up
INAVA	Up
NUF2	Up
IFI44	Up
PLK1	Up
DDX60	Up
RAD51AP1	Up
DEFA1	Up
RNF17	Up
LY6E	Up
ERCC6L	Up
FBXO16	Up
FBXO39	Up
POU2AF1	Up
MAOA	Up
FAM83D	Up
PCLAF	Up
ZCCHC2	Up
SGO1	Up
TEX9	Up
SIGLEC1	Up
LAX1	Up
TRIM6	Up
ZNF215	Up
DAW1	Up
RGS13	Up
OAS2	Up
PRC1	Up
CCDC150	Up
EGF	Up
TEK	Up
CEP128	Up
SPAG5	Up
CIP2A	Up
MPO	Up
TACSTD2	Up
PTX3	Up
SCD	Up
TSHR	Up
MCM4	Up
LEP	Up
RSC1A1	Up
ARHGAP11B	Up
IFIT3	Up
UBE2T	Up
ZBTB32	Up
XAF1	Up
SKA1	Up
NCAPG2	Up
CDCA7	Up
PIMREG	Up
H2BC15	Up
NNMT	Up
ZNF608	Up
MAB21L3	Up
BPI	Up
LMAN1	Up

CCNB1	Up
MCM4	Up
PIMREG	Up
DEFA1B	Up
CHEK1	Up
MND1	Up
NUSAP1	Up
SIK1B	Up
LOC112268355	Up
ZNF215	Up
HSP90B1	Up
GTSE1	Up
SEC11C	Up
SLC35F2	Up
DOC2B	Up
FBXO43	Up
CCNE1	Up
PSAT1	Up
CCDC150	Up
OLR1	Up
NCAPG2	Up
TEDC2	Up
PLK1	Up
TIMD4	Up
BHLHB9	Up
SMC2	Up
PHGDH	Up
MCM2	Up
PRRT4	Up
CD38	Up
PRTN3	Up
ANKRD36C	Up
SKA1	Up
PCLAF	Up
TCF19	Up
DDR2	Up
ARHGAP11A	Up
TRAM2	Up
UAP1	Up
HID1	Up
PHKA1	Up
XRCC2	Up
SPATS2	Up
INHBA	Up
WDHD1	Up
FKBP11	Up
LNP1	Up
SLC17A9	Up
TEX9	Up
UBE2T	Up
CRISP3	Up
RECQL4	Up
A2M	Up
PRC1	Up
UHRF1	Up
SGO1	Up
KIF23	Up
CHAC1	Up

OR2A42	Up
OIP5	Up
PLSCR4	Up
ITM2C	Up
COBLL1	Up
AMPD1	Up
OR2B6	Up
IGF2BP3	Up
CADM1	Up
GGH	Up
MX1	Up
BRCA2	Up
PPIAL4D	Up
SNURF	Up
FCRL4	Up
HERC5	Up
TENT5C	Up
TCN1	Up
CCNE1	Up
MRAP2	Up
MYO1D	Up
CYP7A1	Up
IRF4	Up
NBPF20	Up
MAP3K15	Up
KIAA1958	Up
ACOXL	Up
BHLHE22	Up
TCTEX1D1	Up
CA12	Up
MYRFL	Up
GBP1	Up
RGPD6	Up
TK1	Up
PKHD1L1	Up
H2BC9	Up
TRPV3	Up
DGKH	Up
GINS4	Up
COL4A4	Up
CRACD	Up
HESX1	Up
MED12L	Up
ZNF98	Up
FAM47E-STBD1	Up
TRAM2	Up
SERPINB10	Up
LAMC1	Up
TMEM255A	Up
ASAP2	Up
ACER2	Up
OXTR	Up
MME	Up
CAV2	Up
RASGRP3	Up
TMEM92	Up
CHAC1	Up
IGLC7	Up

COL24A1	Up
TP73	Up
SLC39A14	Up
GINS4	Up
MPO	Up
RFC3	Up
INAVA	Up
CDCA7	Up
TAF4B	Up
FBXO5	Up
FAM171A1	Up
LPO	Up
MAD2L1	Up
FCRL5	Up
SLC25A10	Up
CDKN3	Up
SLC1A4	Up
MANEA	Up
KCNQ5	Up
PTTG1	Up
COL4A3	Up
LTF	Up
MTBP	Up
PKMYT1	Up
GSTCD	Up
PLS1	Up
SPAG4	Up
DNMT3B	Up
SLC27A2	Up
PDIA4	Up
ORC6	Up
CDC25C	Up
NAV2	Up
DNAH10	Up
MMP15	Up
EIF2AK3	Up
SLCO5A1	Up
CENPK	Up
CLSPN	Up
HMGB3	Up
STMN1	Up
HLTF	Up
E2F5	Up
CDKN2A	Up
TARM1	Up
ATAD2	Up
FAM72B	Up
FBXO16	Up
FA2H	Up
CENPU	Up
LRP12	Up
WFS1	Up
OIP5	Up
MATN2	Up
ARNT2	Up
PIF1	Up
CEACAM6	Up
OSBPL10	Up

B9D1	Up
PCOLCE2	Up
SPC25	Up
OAS1	Up
RNASE1	Up
SLC27A2	Up
IGSF11	Up
FCRL5	Up
DEFA1B	Up
SPATS2	Up
OSBPL10	Up
CDCA8	Up
CHAC2	Up
UHRF1	Up
ANKRD34B	Up
CLSPN	Up
OR52K1	Up
CEP97	Up
DYNC1I1	Up
WDHD1	Up
MYZAP	Up
TMEM54	Up
GLDN	Up
WDR62	Up
SLC35F2	Up
KCNK9	Up
MYO3B	Up
ATAD2	Up
SLC8A3	Up
CDKN3	Up
CAPN13	Up
TWIST2	Up
TMEM145	Up
NES	Up
LYG2	Up
KIF24	Up
PRRG4	Up
MAOB	Up
TCF19	Up
BAMBI	Up
STMN1	Up
UBE2C	Up
XRCC2	Up
SPATS2L	Up
MMRN1	Up
SH3TC2	Up
CXCL3	Up
ADAM17	Up
CCL8	Up
GBP7	Up
TSSK4	Up
PTTG1	Up
COL17A1	Up
WEE1	Up
ANKRD36C	Up
XK	Up
PLS1	Up
TTLL7	Up

NT5DC2	Up
CCDC39	Up
LAMC1	Up
RPH3A	Up
ZBED6	Up
IL12A	Up
THBS4	Up
SPC24	Up
CTH	Up
GGH	Up
SLC25A4	Up
FEN1	Up
PROB1	Up
DPF3	Up
CDCA8	Up
RAB30	Up
TPRG1	Up
H2BC17	Up
ZNF90	Up
SMIM33	Up
SNURF	Up
SCD	Up
PPAT	Up
HTRA3	Up
AURKB	Up
BCL7A	Up
KCNA2	Up
SUCNR1	Up
DIRAS2	Up
KIF18B	Up
ALDH1A3	Up
DIPK1A	Up
B4GALT2	Up
POLR3G	Up
FUT10	Up
CXCR3	Up
ZWILCH	Up
XBP1	Up
CAP2	Up
AQP3	Up
SEPTIN3	Up
BPI	Up
WDR76	Up
FANCI	Up
DHFR	Up
KIF20B	Up
PDIA5	Up
POLA1	Up
C3orf52	Up
MCM8	Up
TIMELESS	Up
SNX25	Up
FAM3C	Up
TERT	Up
GATD3A	Up
ZGRF1	Up
CCL25	Up
MS4A3	Up

KIF23	Up
ORC6	Up
MCM2	Up
E2F1	Up
MACC1	Up
FAM72B	Up
NBPF10	Up
ZC3HAV1L	Up
ARHGEF35	Up
RHOBTB1	Up
BCL2L15	Up
DYRK3	Up
SERPING1	Up
ETV7	Up
CNTF	Up
NAV2	Up
LPO	Up
CD24	Up
H4C4	Up
CRYM	Up
IFIT2	Up
BEND4	Up
PIR	Up
SLC22A16	Up
CENPU	Up
NID2	Up
GBP4	Up
CARD17	Up
COL1A2	Up
ZNF367	Up
PSAT1	Up
CDC14B	Up
ARHGEF12	Up
CFAP54	Up
BTN3A1	Up
IFIH1	Up
FOXC1	Up
DDR2	Up
ARHGAP11A	Up
NUGGC	Up
FRMD3	Up
DENND5B	Up
MACIR	Up
TMEM98	Up
FAM72D	Up
CDC7	Up
KCNQ5	Up
FAM47E	Up
ZNF705A	Up
IFI6	Up
CIBAR2	Up
PARP14	Up
AURKA	Up
MS4A3	Up
H2BC8	Up
PRRT4	Up
APOL6	Up
GLI1	Up

ARFGEF3	Up
PPFIA4	Up
TMEM97	Up
KIF24	Up
ATAD5	Up
GOLGA8A	Up
BFSP2	Up
FAM47E-STBD1	Up
PALM2AKAP2	Up
DZIP1L	Up
FKBP7	Up
MINAR1	Up
LRP5	Up
WNT5B	Up
ELL2	Up
SIX5	Up
MAGED1	Up
GCSH	Up
SPC25	Up
SUV39H2	Up
GFI1	Up
NDFIP2	Up
AURKA	Up
ITGA9	Up
SGO2	Up
MYO5C	Up
KCNA3	Up
ARHGAP23	Up
SERPINB10	Up
PAICS	Up
ZDBF2	Up
KLRC4	Up
STARD5	Up
GALNT18	Up
MMP1	Up
SACS	Up
LARP1B	Up
CCNF	Up
FIGNL1	Up
SLC12A2	Up
ZNF827	Up
HSPA13	Up
SSPN	Up
EML6	Up
NBPF20	Up
CENPS	Up
SLC18A2	Up
PKHD1L1	Up
H2BC15	Up
LIN9	Up
DAW1	Up
MTMR8	Up
ESR1	Up
BSPRY	Up
ITGA8	Up
WEE1	Up
LOC389831	Up
MSANTD3-TMEFF1	Up

MCM6	Up
MAD2L1	Up
RNASE3	Up
SATB2	Up
ILDR1	Up
ABCB9	Up
WASF1	Up
C12orf75	Up
MANEA	Up
RGS16	Up
NR0B1	Up
IFIT5	Up
OXCT2	Up
BTN1A1	Up
TRIM55	Up
FN1	Up
PARPBP	Up
IL12A	Up
TAF4B	Up
LAMP3	Up
CDC42BPA	Up
NDC80	Up
ITGA9	Up
MGAM2	Up
LONRF3	Up
RAB23	Up
FHL2	Up
SMC2	Up
LOC283710	Up
PTH2R	Up
FBXO5	Up
AFF2	Up
ZBTB16	Up
GALM	Up
DHCR24	Up
SLFN5	Up
FANCI	Up
LHX4	Up
ARNTL2	Up
RAPGEF4	Up
IRAK2	Up
NPY1R	Up
NBPF4	Up
FRZB	Up
SAMD9L	Up
SLC23A1	Up
SLC2A14	Up
OR2B2	Up
EGR4	Up
COL10A1	Up
GUCY1A1	Up
CALHM5	Up
ADAMTS6	Up
NET1	Up
IGHA2	Up
CCDC173	Up
APOBEC3A_B	Up
ABCA1	Up

MARS2	Up
PSG11	Up
AUNIP	Up
CABLES1	Up
RGS16	Up
BCL2L15	Up
SETBP1	Up
FAM149A	Up
CENPJ	Up
TRPV3	Up
BLNK	Up
MACC1	Up
PDLIM4	Up
INCENP	Up
SEL1L3	Up
MPHOSPH9	Up
PARPBP	Up
PNOC	Up
OXCT1	Up
TMEM200A	Up
SLC16A1	Up
RAD54B	Up
IARS1	Up
MTHFD1	Up
NET1	Up
GINS3	Up
PARP2	Up
TUBB1	Up
CCL13	Down
DCANP1	Down
TNNI3	Down
COL4A5	Down
PRDM13	Down
CERKL	Down
KCTD14	Down
REN	Down
CPLX4	Down
OR4D1	Down
AKAIN1	Down

SCN9A	Up
ASS1	Up
TMED7-TICAM2	Up
LCN2	Up
MYB	Up
PRR15	Up
TEDC2	Up
HELB	Up
GBP6	Up
NBPF19	Up
LTBP1	Up
SUV39H2	Up
SH3GL3	Up
DNA2	Up
HPDL	Up
IQUB	Up
SPAG1	Up
NODAL	Up
ANTXRL	Up
CGN	Up
SLC35D3	Up
MYOZ3	Up
FA2H	Up
CFAP43	Up
PCP4L1	Up
RMI2	Up
CD80	Up
NKX3-1	Up
TEX2	Up
PLS3	Up
USP43	Up
SMIM38	Up
FAM72C	Up
CPA2	Up
RHCE	Up
NSG2	Up
UGT2B17	Up
DEPP1	Up
ELL2	Up
SYNJ2	Up
TLCD4	Up
AURKB	Up
BEND7	Up
TRIM22	Up
JADE3	Up
LY6G6C	Up
EBLN2	Up
GBP5	Up
BTBD19	Up
NAT8	Up
LANCL3	Up
PCYT1B	Up
C1orf226	Up
STARD4	Up
SSPN	Up
KLHDC8A	Up
XIRP2	Up
WLS	Up

CENPM	Up
E2F5	Up
LNP1	Up
RNF169	Up
EPAS1	Up
SCN8A	Up
SPC24	Up
FZD4	Up
FBXL13	Up
NAV3	Up
FCGR3B	Up
CCL25	Up
SCN5A	Up
PROM1	Up
SLC12A2	Up
XRN1	Up
BMP8B	Up
ESR1	Up
RAPGEF5	Up
EIF2AK2	Up
MINAR1	Up
SAMD9	Up
TG	Up
KIAA1614	Up
FUT8	Up
DNAH10	Up
MLNR	Up
RIMKLB	Up
TPD52	Up
H2BC5	Up
ATAD5	Up
CCDC80	Up
SGO2	Up
STON1	Up
KAZN	Up
CTNNAL1	Up
SLC18A2	Up
IL21	Up
PIF1	Up
ELOVL7	Up
ARFGEF2	Up
DOCK1	Up
ZWILCH	Up
CYP4F3	Up
RAD54B	Up
NLRP7	Up
AIF1L	Up
MEIOC	Up
NCR1	Up
EYA2	Up
EIF2AK3	Up
MCM8	Up
TUBB1	Up
DHFR	Up
POGLUT2	Up
DZIP1L	Up
CD109	Up
GOLGA8T	Up

PLPPR3	Up
UAP1	Up
IGHM	Up
TFPI	Up
CHIT1	Up
KIF18B	Up
CYP1A1	Up
ARHGEF5	Up
TIMELESS	Up
PHGDH	Up
ZNF724	Up
CDT1	Up
RAB30	Up
FAM151A	Up
PLCB4	Up
ART4	Up
GALNT4	Up
SPATA31C2	Up
SLC39A14	Up
HERC6	Up
WDR76	Up
GOLGA8Q	Up
PRTN3	Up
LRRK2	Up
ZNF684	Up
ITGA8	Up
IL12RB2	Up
SEPTIN4	Up
SLFN12L	Up
RACGAP1	Up
TNIK	Up
GABRR2	Up
TPST1	Up
CENPN	Up
GFI1	Up
USP6NL	Up
LDLR	Up
LRIT2	Up
SORBS1	Up
MAML2	Up
NPR3	Up
PEAR1	Up
CABLES1	Up
HERC2	Up
CD274	Up
CSGALNACT2	Up
PP2D1	Up
IGFL4	Up
CRISP3	Up
VXN	Up
STAT1	Up
TLCD4-RWDD3	Up
LEFTY2	Up
LOC150051	Up
ZBTB38	Up
MYO1B	Up
EMP1	Up
GSTCD	Up

DIPK1A	Up
RASSF6	Up
GPR75	Up
MPHOSPH9	Up
TRIM5	Up
VPS13A	Up
ATP7B	Up
OR6K3	Up
IL18R1	Up
SLFN13	Up
BAAT	Up
H2BC11	Up
PHOSPHO2-KLHL23	Up
ZRANB1	Up
LRRC37A3	Up
FKBP11	Up
PDZD2	Up
KCNA2	Up
MNS1	Up
ZNF788P	Up
PPIAL4G	Up
CXCL10	Up
SPIN4	Up
POLA1	Up
H1-6	Up
RALYL	Up
ANKRD33B	Up
CAPN9	Up
CCNF	Up
SPDL1	Up
KIF20B	Up
SLC51B	Up
BCAR3	Up
FOXD4L5	Up
DUSP13	Up
MASP2	Up
CARMIL1	Up
POMK	Up
AUNIP	Up
BMP6	Up
CDR2L	Up
SPTBN1	Up
LIN9	Up
FUT10	Up
IL1RL2	Up
GYPA	Up
INHBC	Up
ERFE	Up
NPIPB13	Up
COL24A1	Up
PSPH	Up
TYMSOS	Up
SEC14L5	Up
UBQLNL	Up
PRDM13	Up
TSTD2	Up
EZH2	Up
MCF2	Up

JUN	Up
PLEKHM3	Up
PLPP3	Up
PARP9	Up
KNTC1	Up
EPG5	Up
CYP26A1	Up
BTN3A3	Up
HEATR5B	Up
RGPD8	Up
ATP8A1	Up
MAP2	Up
ZBED2	Up
CCDC110	Up
HSPA13	Up
LETM2	Up
FEN1	Up
RTL4	Up
MYLK	Up
SELENOP	Up
RAB39A	Up
PDYN	Up
MSRB3	Up
SLCO5A1	Up
SRGAP2B	Up
ALPK3	Up
RD3L	Up
DPF3	Up
RHPN2	Up
NEXN	Up
ABLIM3	Up
SEPTIN11	Up
PLN	Up
SCN3A	Up
SPAG4	Up
VIT	Up
RNF213	Up
ARHGAP23	Up
SELENOI	Up
CENPJ	Up
ECT2	Up
HSD11B2	Up
LIPH	Up
CHST1	Up
GOLGA6C	Up
FAM72A	Up
DUSP5	Up
IFI27L1	Up
PRKG1	Up
SBF2	Up
GMNN	Up
RTP4	Up
MED13L	Up
VANGL1	Up
NKD1	Up
NDFIP2	Up
CASP8AP2	Up
SLC27A6	Up

BLM	Up
ITGA1	Up
CDK14	Up
BATF2	Up
MYCBP2	Up
KIAA1109	Up
PDE5A	Up
CEP152	Up
STOX2	Up
TASOR2	Up
SPTA1	Up
BIRC6	Up
CRACR2A	Up
SETDB2-PHF11	Up
NCOA7	Up
LHX1	Up
VEGFC	Up
EML6	Up
TUFT1	Up
MYSM1	Up
DNER	Up
EPDR1	Up
FAM149A	Up
SLC2A4	Up
PCGF5	Up
BRIP1	Up
TRHR	Up
SPESP1	Up
SLC4A11	Up
TRIM40	Up
SAP30	Up
HEMGN	Up
GBP3	Up
REV3L	Up
PSMA8	Up
GRB14	Up
CCDC85A	Up
ABCC4	Up
SPATA1	Up
RFC5	Up
TRPC6	Up
LHFPL2	Up
RNF39	Up
GEN1	Up
PPAT	Up
F2R	Up
NCKAP1	Up
NWD1	Up
C18orf25	Up
MMP2	Up
LRRN2	Up
SPARC	Up
PTPRU	Up
TNFRSF19	Up
MYO5C	Up
CALD1	Up
LRP6	Up
CRNDE	Up

NBPF14	Up
HERC3	Up
ROCK2	Up
F13A1	Up
TAAR1	Up
KIR2DS4	Up
SPICE1	Up
ELOVL6	Up
ELF1	Up
DNAJB7	Up
LAPTM4B	Up
IL1R1	Up
STON2	Up
B4GALT5	Up
TMEM252	Up
SLC6A9	Up
ITGB3	Up
ZNF559-ZNF177	Up
OR2A4	Up
STAB2	Up
VWA8	Up
ITGA2	Up
RARRES1	Up
PNMA6F	Up
CPEB4	Up
SLC1A4	Up
LIMS4	Up
PELI1	Up
C3orf52	Up
SOX5	Up
EPB41L5	Up
LATS2	Up
ARG2	Up
NAV1	Up
DNAJC6	Up
HSP90B1	Up
LCA5	Up
SLC16A1	Up
SLC22A15	Up
COL6A3	Up
SIPA1L2	Up
TIMD4	Up
EPHB1	Up
TRIM58	Up
MMD	Up
ARHGAP21	Up
PRKAR2B	Up
NFAT5	Up
PGAP1	Up
ZSCAN5B	Up
STBD1	Up
FAXDC2	Up
BEAN1	Up
MTHFD1	Up
TMEM123	Up
GALNT18	Up
EDEM3	Up
SCIN	Up

KLHL23	Up
MASTL	Up
BVES	Up
INSYN2B	Up
TIPIN	Up
MTFR2	Up
ANO5	Up
FNDC3A	Up
EGR1	Up
FLT1	Up
CTAGE15	Up
DMC1	Up
JAM3	Up
INCENP	Up
USF3	Up
TAFA2	Up
SASS6	Up
CLDN12	Up
CD5L	Up
ZKSCAN7	Up
VNN3	Up
HMGB3	Up
RBM11	Up
A2M	Up
TMSB15A	Up
TCEAL9	Up
PEAK1	Up
NPIPB2	Up
SLC44A1	Up
SH3RF2	Up
DDN	Up
S100A7	Up
CHST4	Up
SEL1L3	Up
MYH11	Up
AMY2A	Up
PKD2	Up
SMCHD1	Up
CHST8	Up
SLA2	Up
PIWIL4	Up
FZD3	Up
CFAP251	Up
KIAA0754	Up
H2BC4	Up
RIC1	Up
BMPR2	Up
CTHRC1	Up
PLCXD2	Up
SEC11C	Up
HNRNPLL	Up
VPS13B	Up
CATSPERE	Up
TCF4	Up
SLC30A3	Up
IRS1	Up
NEK4	Up
FGF2	Up

CTAGE8	Up
PHACTR1	Up
PDP2	Up
DNAJC13	Up
GOLGA6L10	Up
CDK17	Up
OSGIN2	Up
CCDC62	Up
ZNF66	Up
CAMP	Up
PLOD2	Up
SERPINE1	Up
CUBN	Up
MOV10L1	Up
TOM1L1	Up
CLEC5A	Up
RGS6	Up
UBR1	Up
NCOA3	Up
LPIN2	Up
ZC2HC1B	Up
NXF5	Up
KLK10	Up
PARP11	Up
DDAH1	Up
C2CD3	Up
CHMP4C	Up
XCR1	Up
STX17	Up
SMG1	Up
DTHD1	Up
TMEM217	Up
ROR2	Up
KLRC2	Up
MEIS1	Up
BBX	Up
ZBTB43	Up
RICTOR	Up
RESF1	Up
CDO1	Up
ANKIB1	Up
CSGALNACT1	Up
LRP12	Up
PATL1	Up
C5orf47	Up
CILP	Up
NR2C2	Up
PRKCE	Up
KLRC3	Up
VNN1	Up
SPRY1	Up
PRR14L	Up
CDK2	Up
DOK5	Up
STAT2	Up
VSIG10	Up
ABCC11	Up
ATXN7	Up

GPLD1	Up
COCH	Up
NIPA1	Up
NKAIN2	Up
LRRC8B	Up
TAF2	Up
GSDMC	Up
SLC26A8	Up
USP37	Up
BRCA1	Up
SRGAP2C	Up
N4BP2	Up
FAM169A	Up
PAWR	Up
ZNF888	Up
POF1B	Up
BDP1	Up
SCML2	Up
PPP1R2B	Up
CFAP299	Up
NSD2	Up
SLC38A1	Up
CREBRF	Up
ACADM	Up
PPP1R14C	Up
PERP	Up
CSF1	Up
PPM1K	Up
ASTN2	Up
ZSCAN12	Up
ARHGAP6	Up
PRTFDC1	Up
RFC3	Up
SH2D4A	Up
TMEM97	Up
DOK6	Up
CRYBG3	Up
PRUNE2	Up
TCP11L2	Up
BARD1	Up
CXCL13	Up
MEGF9	Up
PRRG1	Up
NEK7	Up
MEP1A	Up
FRK	Up
TXNDC2	Up
CFAP221	Up
BICD1	Up
FAM106A	Up
BPTF	Up
SLCO4C1	Up
SLC38A2	Up
DTX3L	Up
DDX58	Up
ABCC2	Up
SPSB1	Up
BTN2A2	Up

NCAPD3	Up
ZNF365	Up
ACCSL	Up
STXBP5	Up
USP28	Up
RBM43	Up
SLAMF7	Up
HLTF	Up
KCNJ1	Up
BTLA	Up
TNPO1	Up
NRSN1	Up
NR5A2	Up
NUP205	Up
PLGLB2	Up
AMOTL1	Up
FLT3	Up
PAICS	Up
CBX2	Up
FNDC3B	Up
CDCA7L	Up
AMIGO2	Up
PLD6	Up
APP	Up
MAP3K21	Up
RGS18	Up
CCDC39	Up
KIAA0319	Up
LRBA	Up
SESTD1	Up
POGLUT3	Up
DYTN	Up
PIKFYVE	Up
SDE2	Up
CCDC144A	Up
KLB	Up
NCOA2	Up
RSF1	Up
PRKD1	Up
SCG5	Up
LTN1	Up
HSD17B6	Up
KDELR3	Up
SYCE2	Up
FBXO34	Up
DNMT3B	Up
CPEB2	Up
SLC25A4	Up
REXO5	Up
FKBP14	Up
CYS1	Up
CPO	Up
SULT1E1	Up
RIPOR3	Up
RAG1	Up
ADGRF5	Up
ATF3	Up
BRPF3	Up

RNASE2	Up
EHD3	Up
SPIC	Up
AASS	Up
PAG1	Up
VEPH1	Up
GUCY1B1	Up
LYST	Up
ANKRD26	Up
SLFN12	Up
CCR8	Up
LRFN2	Up
KLHL8	Up
ARHGAP5	Up
ZDHHC23	Up
SLCO4A1	Up
SOS1	Up
GP6	Up
FAM8A1	Up
TNFAIP3	Up
C2orf88	Up
SMC1B	Up
ERBB3	Up
RDX	Up
STAP1	Up
AP1M2	Up
BOD1L1	Up
NUAK1	Up
ANGPT1	Up
CRISPLD1	Up
RAB27B	Up
NPIPB9	Up
LOC102723360	Up
HOXB7	Up
KCNK5	Up
SLC25A51	Up
PLEKHS1	Up
HIPK2	Up
PDIA5	Up
NBEA	Up
INO80D	Up
SACS	Up
ZBP1	Up
LATS1	Up
PPP4R4	Up
FAR2	Up
LUC7L2	Up
UNC13B	Up
NBN	Up
INHBE	Up
DCLRE1A	Up
CCDC186	Up
CDIN1	Up
TRPM6	Up
SLC6A3	Up
PHACTR2	Up
SYTL4	Up
PDGFD	Up

CNGA4	Up
PDCD1LG2	Up
MYO6	Up
FCRL6	Up
KCND1	Up
CA3	Up
PTGFR	Up
FANCM	Up
HTR1F	Up
FIGNL1	Up
MAGEB10	Up
ATXN1L	Up
ATP8B4	Up
SOX10	Up
PKMYT1	Up
BLNK	Up
SSX2IP	Up
CCKBR	Up
CKAP2	Up
COL19A1	Up
ALG11	Up
LRP8	Up
GOLGA6L2	Up
ESM1	Up
SPG11	Up
CEP57L1	Up
ANO6	Up
FAT4	Up
DPPA4	Up
GPD2	Up
GINS3	Up
PI4K2B	Up
MED13	Up
TRIM69	Up
SNX25	Up
OR52K2	Up
TAGAP	Up
SYDE2	Up
TCF20	Up
PARD3	Up
SPIN1	Up
GPR180	Up
DONSON	Up
KY	Up
ZGRF1	Up
GCSH	Up
ZNF214	Up
PDE2A	Up
YOD1	Up
BTN3A2	Up
FMN1	Up
ABCG1	Up
CLIC4	Up
GCC2	Up
KMT2C	Up
PDE3A	Up
STK33	Up
PCDHGA11	Up

EIF4G3	Up
CASP10	Up
TFRC	Up
CKS2	Up
EFR3A	Up
BHLHB9	Up
H3C1	Up
KCNJ13	Up
CELA2A	Up
SETX	Up
F3	Up
DHRS9	Up
RP2	Up
TNKS2	Up
DNM3	Up
SQLE	Up
VN1R2	Up
GULP1	Up
PPL	Up
ZSCAN29	Up
LY75-CD302	Up
KCNA3	Up
SAMD12	Up
KLHL24	Up
CPED1	Up
CYP51A1	Up
GFPT1	Up
TLN2	Up
CEP76	Up
EPB41	Up
CD2AP	Up
SLC6A16	Up
MINDY4B	Up
ZNF699	Up
C5	Up
SLC5A3	Up
RAB19	Up
C18orf54	Up
STRBP	Up
CHRNA6	Up
TLDC2	Up
ARL5B	Up
FER	Up
TNFSF12-TNFSF13	Up
LCOR	Up
TAGLN	Down
LIME1	Down
RPS28	Down
CARD9	Down
MARCO	Down
VWCE	Down
C7orf50	Down
SEZ6L	Down
GPR35	Down
MBD3	Down
ANKRD35	Down
CDC42EP1	Down
ZNF358	Down

ID3	Down
TNNI2	Down
IGFBP6	Down
ARPIN-AP3S2	Down
ANTKMT	Down
TSPO	Down
NR4A1	Down
SDSL	Down
GNB1L	Down
CLEC10A	Down
NAPSA	Down
ELOB	Down
USP2	Down
GZMM	Down
SPNS3	Down
DGCR6	Down
TGM3	Down
LENG9	Down
SCAND1	Down
NPDC1	Down
FUT7	Down
NME3	Down
SIVA1	Down
MPPED1	Down
HSPB1	Down
MCRIP1	Down
ZDHHC1	Down
SLC39A4	Down
FAM174C	Down
ATOH8	Down
CDKN1C	Down
S1PR4	Down
SYNC	Down
UPK3A	Down
DPP7	Down
NDUFB7	Down
CD7	Down
ZNF524	Down
JOSD2	Down
OSGIN1	Down
BEGAIN	Down
NAPRT	Down
NDUFS7	Down
OR1F12	Down
MMP17	Down
ATP5F1D	Down
IL4	Down
LYNX1	Down
HBB	Down
LOC102724770	Down
FNDC10	Down
SEMA6B	Down
ZNF703	Down
NOXA1	Down
LYPD2	Down
FAM3D	Down
RNA28SN4	Down
KSR2	Down

VMO1	Down
PGA4	Down
NEURL1	Down
CRIP2	Down
PPP1R17	Down
TPPP3	Down
FABP1	Down
CACNA2D3	Down
CERKL	Down
RNA5S1	Down
RNA5S10	Down
RNA5S11	Down
RNA5S12	Down
RNA5S13	Down
RNA5S14	Down
RNA5S15	Down
RNA5S16	Down
RNA5S17	Down
RNA5S2	Down
RNA5S3	Down
RNA5S4	Down
RNA5S5	Down
RNA5S6	Down
RNA5S7	Down
RNA5S8	Down
MAT1A	Down
ASB5	Down
GPIHBP1	Down
HBA1	Down
SAA2-SAA4	Down
ANKS1B	Down
HBA2	Down
DMRTC1B	Down
CRYAB	Down
