# Supplementary material for: Identification of the potential association between SARS-CoV-2 infection and acute kidney injury based on the shared gene signatures and regulatory network
Source: BMC Infect Dis. 2023 Oct 3;23:655. doi: 10.1186/s12879-023-08638-6 (PMC10548629; doi:10.1186/s12879-023-08638-6)
Supplement: Supplementary file 4 — Supplementary Material 4 [file 12879_2023_8638_MOESM4_ESM.pdf]

Table S1. Protein–protein interaction network

Subnetwork1		Subnetwork2		Subnetwork3	
Gene signature	Interacting protein	Gene signature	Interacting protein	Gene signature	Interacting protein
EGF	ITGB1	RRM2	RRM2B	CUBN	CBLIF
COL6A3	ITGB4		CMPK2		ALB
	CSK		ASF1B		AMN
	LAMB1		MELK		GC
	PLCG1		PLK1		
	PTPN12		RRM1		
	PDGFRA		DTYMK		
	BLK		TYMS		
	VWF		UBE2C		
	CDH1		DTL		
	PDGFRB		ASPM		
	GAB1		CDC20		
	LAMC2		PCNA		
	FGF2		CDK1		
	ERBB3		CHEK1		
	ERBB2		EIF4G1		
	LRIG1				
	EGFR				
	KIT				
	ZFYVE28				
	PTPN2				
	EDNRA				
	P4HB				
	HSP90AA1				
	LCK				
	PTPN11				
	ERBB4				
	GNAI1				
	JAK1				
	CTNNB1				
	FYN				
	PTPRK				
	ADAM12				
	GNAI3				
	PTPN1				
	SRC				
	FGR				
	PTPN3				
	LAMA1				
	GRB2				

	NRG1<br>SHC1<br>ERBIN<br>PIK3R1<br>LYN<br>NTRK1<br>HCK<br>ITGB3<br>YES1				
