# Supplementary material for: Identification of the potential association between SARS-CoV-2 infection and acute kidney injury based on the shared gene signatures and regulatory network
Source: BMC Infect Dis. 2023 Oct 3;23:655. doi: 10.1186/s12879-023-08638-6 (PMC10548629; doi:10.1186/s12879-023-08638-6)
Supplement: Supplementary file 5 — Supplementary Material 5 [file 12879_2023_8638_MOESM5_ESM.pdf]

Table S2. Transcription factor (TF)–gene interaction network

Subnetwork1		Subnetwork2		Subnetwork3	
Gene signature	Transcription factor	Gene signature	Transcription factor	Gene signature	Transcription factor
RRM2	ELK1	RARRES1	MBD2	EGF	MEF2D
	FOSL1		FOSL2		RFX1
	ZNF324		BHLHE40		GATA2
	NRF1		SUZ12		
	DDX20				
	ETV1				
	ETS1				
	SIN3A				
	ZNF394				
	E2F6				
	PHF8				
	CCNT2				
	BCL11B				
	SMARCA5				
	ZEB1				
	ZNF76				
	CBFB				
	IRF1				
	HDAC6				
	ZNF71				
	ZBTB7A				
	KDM5B				
	NR2F1				
	ZNF644				
	ZFP64				
	ZNF384				
	INSM2				
	ZNF2				
	TEAD3				
	HMGN3				
	RERE				
	TRIM24				
	PML				
	THRB				
	GLI4				
	REST				
	MBD1				
	SOX5				
	ELF3				
	TFDP1				

	NFYC<br>TAF7<br>MLX<br>SAP30<br>POLR2H<br>GATA4<br>SREBF2<br>HIC1<br>E2F5<br>BCL11A<br>ZNF335<br>ZNF197<br>BCL6<br>MXI1<br>ZNF146<br>POLR2A<br>ZNF610<br>KLF9<br>ZNF24<br>MLLT1<br>ZFP37<br>SMAD5<br>CTCF<br>HDAC2				
