# Supplementary material for: Identification of the potential association between SARS-CoV-2 infection and acute kidney injury based on the shared gene signatures and regulatory network
Source: BMC Infect Dis. 2023 Oct 3;23:655. doi: 10.1186/s12879-023-08638-6 (PMC10548629; doi:10.1186/s12879-023-08638-6)
Supplement: Supplementary file 6 — Supplementary Material 6 [file 12879_2023_8638_MOESM6_ESM.pdf]

Table S3. Gene-miRNA interaction network

Subnetwork1		Subnetwork2	
Gene signatures	miRNA	Gene signatures	miRNA
RRM2	hsa-mir-665	CUBN	hsa-mir-140-5p
EGF	hsa-mir-1273e		hsa-mir-520g-3p
	hsa-mir-6807-5p		hsa-mir-520h
	hsa-mir-6840-3p		hsa-mir-607
	hsa-let-7a-5p		hsa-mir-130b-5p
	hsa-let-7b-5p		hsa-mir-3652
	hsa-let-7c-5p		hsa-mir-4430
	hsa-let-7d-5p		hsa-mir-3135b
	hsa-let-7e-5p		hsa-mir-3973
	hsa-let-7f-5p		hsa-mir-4760-3p
	hsa-mir-17-5p		hsa-mir-5590-5p
	hsa-mir-20a-5p		
	hsa-mir-24-3p		
	hsa-mir-26a-5p		
	hsa-mir-30a-5p		
	hsa-mir-93-5p		
	hsa-mir-98-5p		
	hsa-mir-100-5p		
	hsa-mir-101-3p		
	hsa-mir-106a-5p		
	hsa-mir-30c-5p		
	hsa-mir-30d-5p		
	hsa-mir-34a-5p		
	hsa-mir-211-5p		
	hsa-let-7g-5p		
	hsa-let-7i-5p		
	hsa-mir-30b-5p		
	hsa-mir-149-5p		
	hsa-mir-186-5p		
	hsa-mir-155-5p		
	hsa-mir-106b-5p		
	hsa-mir-30e-5p		
	hsa-mir-342-3p		
	hsa-mir-20b-5p		
	hsa-mir-323b-5p		
	hsa-mir-484		
	hsa-mir-485-5p		
	hsa-mir-202-3p		
	hsa-mir-193b-3p		
	hsa-mir-526b-3p		
	hsa-mir-519d-3p		

	hsa-mir-582-5p<br>hsa-mir-425-5p<br>hsa-mir-33a-3p<br>hsa-mir-223-5p<br>hsa-mir-127-5p<br>hsa-mir-149-3p<br>hsa-mir-423-5p<br>hsa-mir-940<br>hsa-mir-1225-3p<br>hsa-mir-1233-3p<br>hsa-mir-1294<br>hsa-mir-1304-5p<br>hsa-mir-302f<br>hsa-mir-664a-5p<br>hsa-mir-1321<br>hsa-mir-1827<br>hsa-mir-3169<br>hsa-mir-3184-5p<br>hsa-mir-3188<br>hsa-mir-4316<br>hsa-mir-4328<br>hsa-mir-3609<br>hsa-mir-4419a<br>hsa-mir-4433a-3p<br>hsa-mir-548ah-5p<br>hsa-mir-4458<br>hsa-mir-4459<br>hsa-mir-3689d<br>hsa-mir-4500<br>hsa-mir-4510<br>hsa-mir-4515<br>hsa-mir-4534<br>hsa-mir-4520-5p<br>hsa-mir-3975<br>hsa-mir-4649-3p<br>hsa-mir-4668-3p<br>hsa-mir-219b-3p<br>hsa-mir-4672<br>hsa-mir-4700-3p<br>hsa-mir-4722-5p<br>hsa-mir-4728-5p<br>hsa-mir-3064-5p<br>hsa-mir-4739<br>hsa-mir-4755-3p		

	hsa-mir-4756-5p<br>hsa-mir-4768-3p<br>hsa-mir-2467-5p<br>hsa-mir-4794<br>hsa-mir-4796-3p<br>hsa-mir-4802-5p<br>hsa-mir-5006-5p<br>hsa-mir-6083<br>hsa-mir-6127<br>hsa-mir-6129<br>hsa-mir-6130<br>hsa-mir-6133<br>hsa-mir-6504-5p<br>hsa-mir-410-5p<br>hsa-mir-494-5p<br>hsa-mir-655-5p<br>hsa-mir-2276-5p<br>hsa-mir-6765-5p<br>hsa-mir-6768-5p<br>hsa-mir-6778-3p<br>hsa-mir-6785-5p<br>hsa-mir-6795-3p<br>hsa-mir-6808-5p<br>hsa-mir-6826-3p<br>hsa-mir-6851-5p<br>hsa-mir-6873-5p<br>hsa-mir-6883-5p<br>hsa-mir-6884-5p<br>hsa-mir-6887-3p<br>hsa-mir-6888-5p<br>hsa-mir-6893-5p<br>hsa-mir-6894-5p<br>hsa-mir-7843-3p<br>hsa-mir-8068<br>hsa-mir-8082		
