# Supplementary material for: Identification of the potential association between SARS-CoV-2 infection and acute kidney injury based on the shared gene signatures and regulatory network
Source: BMC Infect Dis. 2023 Oct 3;23:655. doi: 10.1186/s12879-023-08638-6 (PMC10548629; doi:10.1186/s12879-023-08638-6)
Supplement: Supplementary file 7 — Supplementary Material 7 [file 12879_2023_8638_MOESM7_ESM.pdf]

Table S4. TF-miRNA coregulatory network

Subnetwork1			Subnetwork2		
Gene signatures	Transcription factor	miRNA	Gene signatures	Transcription factor	miRNA
COL6A3	ZBTB6<br>SMAD3<br>NFIC	hsa-miR-127-5p<br>hsa-miR-130b<br>hsa-miR-133a<br>hsa-miR-133b<br>hsa-miR-143<br>hsa-miR-152<br>hsa-miR-181a<br>hsa-miR-181c<br>hsa-miR-181d<br>hsa-miR-198<br>hsa-miR-202<br>hsa-miR-28-3p<br>hsa-miR-29a<br>hsa-miR-29b<br>hsa-miR-29c<br>hsa-miR-301a<br>hsa-miR-301b<br>hsa-miR-345<br>hsa-miR-508-5p<br>hsa-miR-509-3p<br>hsa-miR-518d-5p<br>hsa-miR-520a-5p<br>hsa-miR-525-5p<br>hsa-miR-543<br>hsa-miR-562<br>hsa-miR-568<br>hsa-miR-605<br>hsa-miR-613<br>hsa-miR-617<br>hsa-miR-634<br>hsa-miR-767-5p	RRM2<br>EGF<br>RARRES1	MAX<br>E2F1<br>TP53<br>TFAP2C<br>TFAP2A<br>TCF4<br>TAL1<br>RXRA<br>REST<br>PGR<br>NFYC<br>NFYA<br>NFKB2<br>NFKB1<br>MYC<br>MXI1<br>CLEC5A<br>JUN<br>GABPA<br>FOXA2<br>FOXA1<br>ESR1<br>CEBPB	hsa-let-7b<br>hsa-miR-219-2-3p<br>hsa-miR-30a<br>hsa-miR-30b<br>hsa-miR-30c<br>hsa-miR-30d<br>hsa-miR-30e<br>hsa-miR-561
